# Integration of the tricarboxylic acid (TCA) cycle with cAMP signaling and Sfl2 pathways in the regulation of CO_2_ sensing and hyphal development in *Candida albicans*

**DOI:** 10.1371/journal.pgen.1006949

**Published:** 2017-08-07

**Authors:** Li Tao, Yulong Zhang, Shuru Fan, Clarissa J. Nobile, Guobo Guan, Guanghua Huang

**Affiliations:** 1 State Key Laboratory of Mycology, Institute of Microbiology, Chinese Academy of Sciences, Beijing, China; 2 University of Chinese Academy of Sciences, Beijing, China; 3 Department of Molecular and Cell Biology, University of California, Merced, California, United States of America; University College Dublin, IRELAND

## Abstract

Morphological transitions and metabolic regulation are critical for the human fungal pathogen *Candida albicans* to adapt to the changing host environment. In this study, we generated a library of central metabolic pathway mutants in the tricarboxylic acid (TCA) cycle, and investigated the functional consequences of these gene deletions on *C*. *albicans* biology. Inactivation of the TCA cycle impairs the ability of *C*. *albicans* to utilize non-fermentable carbon sources and dramatically attenuates cell growth rates under several culture conditions. By integrating the Ras1-cAMP signaling pathway and the heat shock factor-type transcription regulator Sfl2, we found that the TCA cycle plays fundamental roles in the regulation of CO_2_ sensing and hyphal development. The TCA cycle and cAMP signaling pathways coordinately regulate hyphal growth through the molecular linkers ATP and CO_2_. Inactivation of the TCA cycle leads to lowered intracellular ATP and cAMP levels and thus affects the activation of the Ras1-regulated cAMP signaling pathway. In turn, the Ras1-cAMP signaling pathway controls the TCA cycle through both Efg1- and Sfl2-mediated transcriptional regulation in response to elevated CO_2_ levels. The protein kinase A (PKA) catalytic subunit Tpk1, but not Tpk2, may play a major role in this regulation. Sfl2 specifically binds to several TCA cycle and hypha-associated genes under high CO_2_ conditions. Global transcriptional profiling experiments indicate that Sfl2 is indeed required for the gene expression changes occurring in response to these elevated CO_2_ levels. Our study reveals the regulatory role of the TCA cycle in CO_2_ sensing and hyphal development and establishes a novel link between the TCA cycle and Ras1-cAMP signaling pathways.

## Author summary

Energy metabolism through the TCA cycle and mitochondrial electron transport are critical for the human fungal pathogen *Candida albicans* to survive and propagate in the host. This is, in part, due to the fact that *C*. *albicans* is a Crabtree-negative species, and thus exclusively uses respiration when oxygen is available. Here, we investigate the roles of the TCA cycle in hyphal development and CO_2_ sensing in *C*. *albicans*. Through the use of ATP and the cellular signaling molecule CO_2_, the TCA cycle integrates with the Ras1-cAMP signaling pathway, which is a central regulator of hyphal growth, to govern basic cellular biological processes. Together with Efg1, a downstream transcription factor of the cAMP signaling pathway, the heat shock factor-type transcription regulator Sfl2 controls CO_2_-induced hyphal growth in *C*. *albicans*. Deletion of *SFL2* results in the loss of global transcriptional responses under elevated CO_2_ levels. Our study indicates that the TCA cycle not only occupies the central position of cellular metabolism but also regulates other biological processes such as CO_2_ sensing and hyphal development through integration with the Ras1-cAMP signaling pathway in *C*. *albicans*.

## Introduction

The ubiquitous tricarboxylic acid (TCA) cycle is a central pathway for the metabolism of carbon sources, lipids, and amino acids, and provides a major energy source for the cell under aerobic conditions. It is composed of a set of enzymes, which are required for the generation of NADH and FADH_2_ electron donors for use in the electron transport chain (ETC). The TCA cycle is also involved in the regulation of a wide range of other biological processes [1] and it is known that mutations in enzymes of the TCA cycle are associated with several neurological disorders and cancers in humans [2]. Due to its importance in energy metabolism and cellular functions, the TCA cycle has been intensively investigated in many different organisms including microbes, humans, plants, and model organisms [3–6]. Little is known, however, about the roles of the TCA cycle in the context of the biology and pathogenesis of the human fungal pathogen *Candida albicans*.

*C*. *albicans* causes not only superficial diseases, but also systemic and disseminated infections in immunocompromised individuals [7, 8]. It has the capacity to colonize virtually every human tissue, but typically exists as a benign commensal in the mouth, gut, and genitourinary tracts of healthy adults [8]. Metabolic plasticity is critical for both the pathogenic and commensal life styles of this fungus. Lorenz and Fink (2001) demonstrated that the glyoxylate cycle regulates macrophage phagocytosis and virulence of *C*. *albicans*, whereby disruption of the glyoxylate pathway prevents the growth of *C*. *albicans* inside macrophages by blocking nutrient availability [9]. During mucosal infections and invasive growth, the TCA cycle and fatty acid β-oxidation-related genes are upregulated [10, 11]. White and opaque cells, which represent two heritable cell types and exhibit distinct virulence profiles in mucosal and systemic infections, differ in their metabolic profiles [12]. Opaque cells, which adopt an oxidative metabolic profile, are more virulent in mucosal infections, whereas white cells, which adopt a fermentative metabolic profile, are more virulent in systemic infections [12, 13]. In addition, a recent proteomics study indicates that the TCA cycle is involved in the control of antifungal tolerance and biofilm formation [14].

The ability to form hyphae is another important biological feature of *C*. *albicans* [15, 16]. The mitogen-activated protein kinase (MAPK) and cAMP signaling pathways are two major players in the control of hyphal growth in *C*. *albicans* [15–17]. Indeed, deletion of *RAS1*, which encodes a small GTPase upstream of the two pathways, leads to attenuation in virulence and defects in hyphal growth under specific culture conditions [18, 19]. Deletion of *CYR1*, which encodes the sole adenylyl cyclase in *C*. *albicans*, completely blocks hyphal growth and leads to a complete loss of infectivity [20]. We recently found that both the catalytic and regulatory subunits of the cAMP-dependent protein kinase A (PKA) are not essential for cell viability in *C*. *albicans* [21, 22]. However, similar to the disruption of *CYR1*, inactivation of the catalytic subunit of PKA by generation of a *tpk2/tpk2 tpk1/tpk1* double mutant, completely blocked hyphal development [22]. The two isoforms of the PKA catalytic subunit, Tpk1 and Tpk2 play redundant and distinct roles in a number of biological processes, such as filamentous growth and responses to cellular stresses [23, 24]. Transcription factors Efg1 and Flo8 are downstream of the cAMP signaling pathway and are essential for filamentous growth under a number of conditions [25, 26]. It is also known that alterations of the cAMP signaling pathway have a remarkable influence on the transcriptional profile of metabolism [22, 27]. Moreover, the activation of the Ras1-cAMP signaling pathway is associated with increased cellular ATP levels and mitochondrial activity in both *C*. *albicans* and *Saccharomyces cerevisiae* [28, 29].

Host-related environmental cues, such as temperature and CO_2_, are important factors in regulating morphological transitions in *C*. *albicans* [15, 30]; for example, high temperatures and elevated levels of CO_2_ promote hyphal growth. There is evidence in support of the idea that cAMP-dependent and -independent pathways are involved in the regulation of CO_2_-induced hyphal growth [31], however the cAMP-independent pathway is yet to be identified. It has been shown that the AGC kinase Sch9 is involved in the regulation of hypoxia and CO_2_ sensing through the control of the transcription factors Czf1 and Ace2 and lipid/Pkh1/2 signaling in *C*. *albicans* [32–34]. The bZIP transcription factor Rca1 regulates the expression of the carbonic anhydrase-encoding gene *NCE103*, which may represent a cAMP-independent pathway of CO_2_ sensing in *C*. *albicans* [35].

Sfl1 and Sfl2, two conserved heat shock factor-type transcription factors, function antagonistically to control morphological transitions in *C*. *albicans* [36–40]. Sfl1 represses filamentous growth, whereas Sfl2 acts as a positive regulator of filamentous growth. Sfl1 and Sfl2 can also act as both transcriptional repressors and activators of certain target genes. Together, Sfl1 and Sfl2 coordinately regulate hyphal development by controlling the expression of hyphal specific genes (HGS) and other regulators, such as Efg1 and Ndt80, in *C*. *albicans* [40].

**Fig 1:**
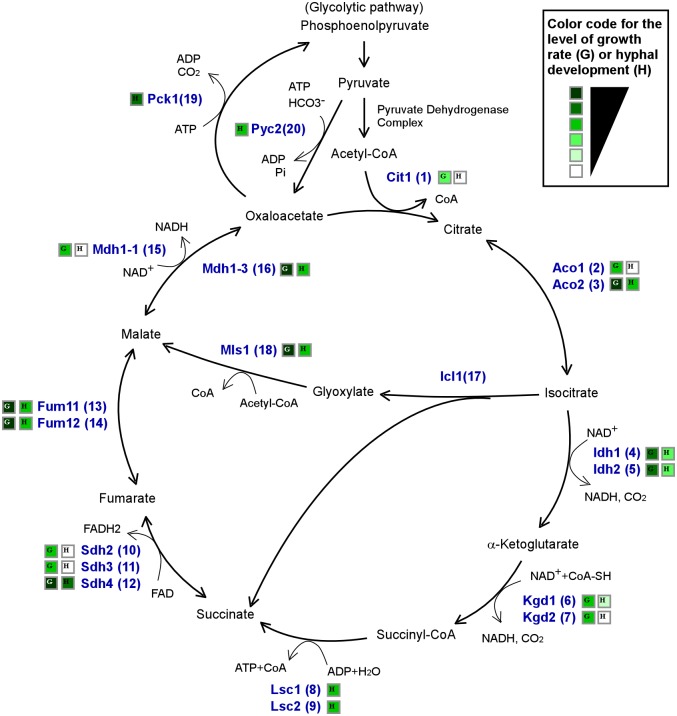
Schematic diagram of the TCA cycle, glyoxylate bypass, and glycolytic pathway in *C*. *albicans*. Metabolic enzymes are indicated by numbers 1 to 20. (1) Citrate synthase, Cit1. (2 and 3) Aconitase, Aco1, Aco2. (4 and 5) Isocitrate dehydrogenase, Idh1, Idh2. (6 and 7) α-Ketoglutarate dehydrogenase, Kgd1, Kgd2. (8 and 9) Succinate-CoA ligase, Lsc1, Lsc2. (10, 11, and 12) Succinate dehydrogenase, Sdh2, Sdh3, Sdh4. (13 and 14) Fumarate hydratase, Fum11, Fum12. (15) Malate dehydrogenase, Mdh1-1. (16) Malate dehydrogenase, Mdh1-3. (17) Isocitrate lyase, Icl1. (18) Malate synthase, Mls1. (19) Phosphoenolpyruvate carboxykinase, Pck1. (20) Pyruvate carboxylase, Pyc2. Color codes for the levels of cell growth rates (G, according to the data of YPD medium at 37°C, S2 and S3 Figs) and hyphal development (H, according to the data of YPD + serum medium at 37°C, S4 Fig) are also indicated.

As a Crabtree-negative and commensal organism, it is critical for *C*. *albicans* to control metabolic and transcriptional processes to adapt to the host environment. As shown in Fig 1, eight enzymes encoded by fifteen genes are involved in the TCA cycle, and isocitratelyase (Icl1), malate synthase (Mls1), and malate dehydrogenase (Mdh1-3) function in the glyoxylate bypass, for a total eighteen genes involved in this major metabolic pathway in *C*. *albicans*. To systemically characterize the roles of the TCA cycle and the glyoxylate bypass in adaption to the host environment, we generated nineteen deletion mutants of the related genes. Our results indicate that through the coordination of the cAMP signaling pathway and the heat shock-type transcription factor Sfl2, the TCA cycle regulates hyphal development and CO_2_ sensing in *C*. *albicans*.

## Results

### Role of the TCA cycle in the utilization of nonfermentable carbon sources

To systemically investigate the biological functions of the TCA cycle and the glyoxylate bypass in *C*. *albicans*, we generated 17 homozygous deletion mutants of genes encoding related metabolic enzymes using traditional fusion PCR strategies (Fig 1) [41]. In addition, we also deleted *PCK1* and *PYC2*, which encode the phosphoenolpyruvate carboxykinase and pyruvate carboxylase, respectively, and control the cellular level of oxaloacetate, an intermediate of the TCA cycle. Since the TCA cycle is the central pathway for carbon and cellular energy metabolism, we examined the growth of these mutants on YNB media containing different carbon sources using serial dilution assays. As shown in S1 Fig, mutants of *CIT1*, *ACO1*, and *MDH1-1*, encoding citrate synthase, aconitase, and malate dehydrogenase, respectively, had no obvious growth on all tested media. Deletion of α-ketoglutarate dehydrogenase-encoding genes, *KGD1* and *KGD2*, and succinate dehydrogenase-encoding genes, *SDH2* and *SDH3*, decreased growth rates on fermentable carbon source-containing media (YNB + glucose, GlcNAc, sucrose, and fructose) but completely blocked cell growth on YNB, YNB + amino acids, YNB + glycerol, or YNB + ethanol media. Deletion of the isocitrate dehydrogenase-encoding genes *IDH1* and *IDH2*, the putative succinate dehydrogenase-encoding gene *SDH4*, and the fumarate hydratase-encoding gene *FUM12* decreased growth on YNB, YNB + amino acids, YNB + glycerol, and YNB + ethanol media. Inactivation of the TCA cycle resulted in the increased activity of the glyoxylate bypass and pyruvate carboxylase. Q-RT-PCR assays indicate that compared to that in the WT control, the expression levels of *ICL1* and *MLS1* were elevated 1.5 to 3.0-fold in the *idh1/idh1* and *idh2/idh2* mutants and the expression of *PYC2* was increased in the *cit1/cit1* mutant 1.5 to 3.0-fold. However, deletion of *ACO2* and the glyoxylate bypass-specific enzyme encoding genes *MLS1* or *MDH1-3* had no obvious effects on the utilization of different carbon sources. To further quantitate the growth rates of the mutants, we cultured the strains in several liquid medium conditions at both 30°C and 37°C. The growth curves are presented in S2 and S3 Figs and the growth abilities are summarized in S1 Table. Our results indicate that the TCA cycle, but not the glyoxylate bypass, plays a critical role in the utilization of both fermentable and nonfermentable carbon sources for energy metabolism in *C*. *albicans*.

### The TCA cycle regulates hyphal development in air and in elevated levels of CO_2_

Hyphal development is one of the most important features of *C*. *albicans* and is associated with virulence [16]. Taking advantage of the TCA cycle mutants generated in this study, we performed hyphal growth assays under twelve culture conditions in air and in the presence of 5% CO_2_ (Table 1). All TCA and glyoxylate cycle-related mutants were able to grow on Spider and YPD media, although some mutants exhibited a reduced growth rate (Table 1 and S1 Table, S2 and S3 Figs). Therefore, neither the TCA nor glyoxylate cycle is essential for cell growth in *C*. *albicans* in rich media or in the presence of all essential nutrients.

**Table 1:** Effect of the TCA gene disruption on hyphal growth of *C*. *albicans* on solid media.

Strain	Air							5% CO_2_				
	Lee’s Glucose		Lee’s GlcNAc		YPD	Spider	YPD+serum	Lee’s Glucose		Lee’s GlcNAc		YPD
	30°C	37°C	30°C	37°C	37°C	37°C	37°C	30°C	37°C	30°C	37°C	37°C
WT	+	++	++++	+++++	-	++++	+++	+++	++++	+++++	++++++	++
*cit1/cit1* (1)	G.d.	G.d.	G.d.	G.d.	-	++	-	G.d.	G.d.	G.d.	G.d.	-
*aco1/aco1* (2)	G.d.	G.d.	G.d.	G.d.	-	++	-	G.d.	G.d.	G.d.	G.d.	-
*aco2/aco2* (3)	+	++	++++	+++++	++	+++	+++	+++	++++	+++++	++++++	+++
*idh1/idh1* (4)	-	+	+	+++	-	++	++	+	++	++	++++	++
*Idh2/idh2* (5)	-	+	+	+++	-	+	++	+	++	++	++++	++
*kgd1/kgd1* (6)	-	+	G.d.	G.d.	-	++++	+	-	+	G.d.	G.d.	-
*Kgd2/kgd2* (7)	-	+	G.d.	G.d.	-	++++	-	-	+	G.d.	G.d.	-
*lsc1/lsc1* (8)	++	+++	++++	++++	-	++++	+++	+++	++++	+++++	++++++	+++
*lsc2/lsc2* (9)	++	+++	++++	++++	-	++++	+++	+++	++++	+++++	++++++	+++
*sdh2/sdh2* (10)	-	++	-	-	-	+++	-	-	++	-	-	-
*sdh3/sdh3* (11)	-	+	-	-	-	++	-	-	+	-	-	-
*sdh4/sdh4* (12)	-	++	+	++++	+	+++	++++	-	+++	++	++++	+++
*fum11/fum11* (13)	+	++	+++	++++	-	+++	+++	+++	++++	+++++	++++++	++
*fum12/fum12* (14)	+	++	++	++++	+	++++	+++	++	++++	++++	++++++	++
*mdh1-1/mdh1-1* (15)	-	-	G.d.	G.d.	-	+	-	-	-	G.d.	G.d.	-
*mdh1-3/mdh1-3* (16)	+	++	+++	++++	++	++++	+++	+++	++++	++++	++++++	+++
*mls1/mls1* (18)	+	++	++++	+++++	+++	++++	+++	+++	++++	+++++	++++++	+++
*pck1/pck1*(19) *pyc2/pyc2*(20)	NA NA	++ G.d.	NA NA	+++++ ++++	+++ +	++++ ++	++++ +++	NA NA	++++ -	NA NA	++++++ +++++	++++ ++

G. d.: Growth defect. The number of “+” signs, indicates the degree of hyphal growth. “-”indicates that no hyphal growth was observed. The degree of hyphal growth was defined according to both the length and percentage of filamentous cells. Cells were initially grown on YPD plates at 30 Celsius degree for three days. Single colonies were resuspended and replated on different media for hyphal development. Incubation time: 5 days on Lee’s glucose or Lee’s GlcNAc medium, 3 days on YPD, YPD+serum, or Spider medium. NA, not available.

#### Hyphal development of the TCA cycle mutants in air

Serum is a potent host-related hyphal inducer. On YPD + serum plates at 37°C, the *cit1/cit1*, *aco1/aco1*, *kgd2/kgd2*, *sdh2/sdh2*, *sdh3/sdh3*, and *mdh1-1/mdh1-1* mutants exhibited defects in hyphal growth, whereas the *idh1/idh1*, *idh2/idh2*, *kgd1/kgd1*, *lsc1/lsc1*, *lsc2/lsc2*, *mdh1-3/mdh1-3*, *mls1/mls1*, and *pyc2/pyc2* mutants showed more moderate defect (S4 Fig). Deletion of *SDH4* and *PCK1* promoted hyphal growth under the same culture condition. The mutants exhibited a similar ability of hyphal development in liquid YPD + 10% serum medium. When cultured on Spider media in air at 37°C, the WT and all TCA cycle mutants underwent hyphal growth, although the *cit1/cit1*, *aco1/aco1*, *idh1/idh1*, *idh2/idh2*, and *mdh1-1/mdh1-1* mutants exhibited relatively weak hyphal growth under these conditions (Table 1). On Lee’s glucose medium, a number of TCA mutants, including *kgd1/kgd1*, *kgd2/kgd2*, and *mdh1-1/mdh1-1*, exhibited defects in hyphal growth at both 30°C and 37°C, and the *idh1/idh1*, *idh2/idh2*, and *sdh4/sdh4* mutants showed reduced hyphal growth on Lee’s GlcNAc medium. A detailed summary of hyphal growth assays under different culture conditions is presented in Table 1. There was an overall correlation between the cell growth rate and the ability to form hyphae (Fig 1 and S1 Table).

#### Hyphal development of the TCA cycle mutants in 5% CO_2_

**Fig 2:**
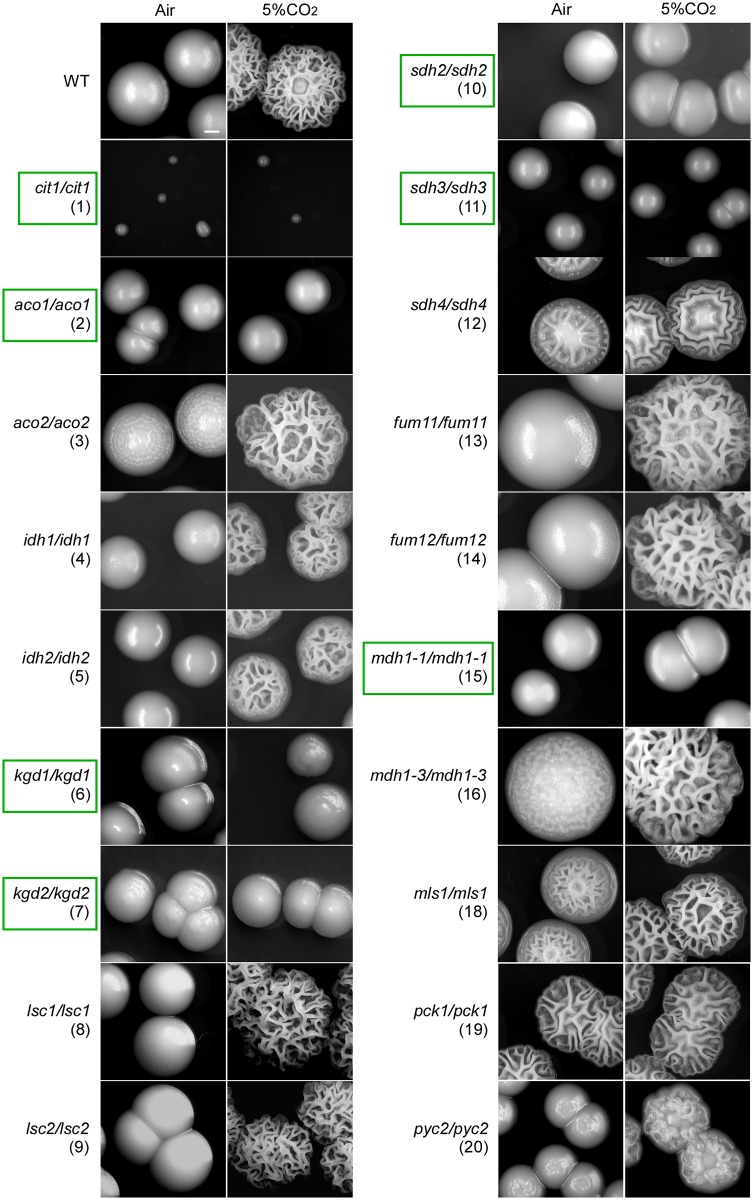
Hyphal development of the TCA and glyoxylate cycle mutants of *C*. *albicans* in air and 5% CO_2_. Green boxes highlight the strains that exhibited hyphal growth defects in 5% CO_2_. The control strain (WT) is SN152. Cells were plated onto YPD medium plates and incubated at 37°C in air or in 5% CO_2_ for three days. Scale bar, 1 mm. To verify the hyphal growth abilities of the mutants, cellular morphologies were examined and presented in S5 Fig. The robustness of hyphal growth is presented in Table 1.

CO_2_ is also a host-related environmental cue and a potent inducer of hyphal growth in *C*. *albicans* [42]. Endogenous CO_2_ is produced at high levels by the TCA cycle and may function as an inducer of hyphal growth or as an intercellular signal in *C*. *albicans* [43]. We next examined the abilities of the TCA mutants to form hyphae in response to an elevated CO_2_ level (5%). On all tested media, this elevated CO_2_ level had no notable effects on cell growth of the TCA cycle mutants. For example, on Lee’s glucose and Lee’s GlcNAc media, the TCA cycle mutants, such as *cit1/cit1* and *aco1/aco1*, exhibited similar growth defects in 5% CO_2_ as they did in air (Table 1 and Fig 2). In the presence of 5% CO_2_, the WT and a portion of mutants (such as *aco2/aco2* and *fum11/fum11*) showed a general increase in hyphal formation on Lee’s glucose and GlcNAc media, whereas the *cit1/cit1*, *aco1/aco1*, *kgd1/kgd1*, *kgd2/kgd2*, *sdh2/sdh2*, *sdh3/sdh3*, *sdh4/sdh4*, and *mdh1-1/mdh1-1* mutants exhibited similar levels of hyphal growth as they did in air at both 30°C and 37°C (Table 1).

When plated and cultured on YPD medium for three days in air at 37°C, the WT and most TCA mutants did not undergo hyphal growth, whereas the *aco2/aco2*, *sdh4/sdh4*, *mls1/mls1*, and *mdh1-3/mdh1-3* mutants formed wrinkled colonies and displayed hyphal growth (Fig 2 and S5 Fig), suggesting that Aco2, Sdh4, Mls1, and Mdh1-3 function as negative regulators of hyphal growth under this culture condition. Of note, *MLS1* and *MDH1-3* are glyoxylate cycle-specific genes. In the presence of 5% CO_2_, the WT, *aco2/aco2*, *idh1/idh1*, *idh2/idh2*, *sdh4/sdh4*, *fum11/fum11*, *fum12/fum12*, *mls1/mls1*, *mdh1-3/mdh1-3*, *lsc1/lsc1*, and *lsc2/lsc2* mutants developed clear hyphae (Fig 2 and S5 Fig). However, similar to the cultures in air, the *cit1/cit1*, *aco1/aco1 kgd1/kgd1*, *kgd2/kgd2*, *sdh2/sdh2*, *sdh3/sdh3*, and *mdh1-1/mdh1-1* mutants did not undergo hyphal growth in 5% CO_2_ (Fig 2 and S5 Fig), suggesting that the metabolic enzymes encoded by these genes are required for CO_2_-induced hyphal growth under this culture condition. As expected, the reconstituted strains of the corresponding TCA cycle genes displayed hyphal growth at similar levels to that of the WT control in air and in 5% CO_2_ (S6 Fig). Pck1 and Pyc2, however, were not required for CO_2_-induced hyphal growth. Taken together, disruption of the TCA cycle had a remarkable effect on CO_2_-induced hyphal growth (Fig 2, S5 Fig and Table 1), suggesting that this cycle may function in both producing and sensing CO_2_ in *C*. *albicans*.

### CO_2_ induces the transcriptional expression of TCA cycle and Ras1-cAMP pathway-related genes

**Fig 3:**
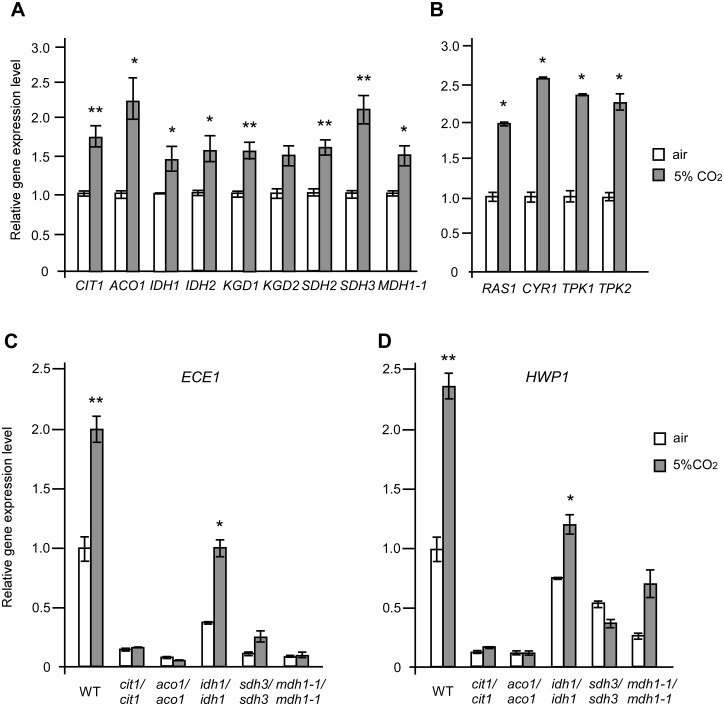
Transcriptional levels of TCA cycle, Ras1-cAMP signaling-related, and hyphal-specific genes in air and 5% CO_2_. (A) Relative expression levels of TCA cycle-related genes, *CIT1*, *ACO1*, *IDH1*, *IDH2*, *KGD1*, *KGD2*, *SDH2*, *SDH3*, and *MDH1-1* in the WT (SN152). (B) Relative expression levels of Ras1-cAMP signaling pathway-related genes *RAS1*, *CYR1*, *TPK1*, and *TPK2* in the WT (SN152). (C and D) Relative expression levels of *ECE1* and *HWP1*. Cells of the control strain (WT, SN152) or indicated mutants were grown on YPD medium plates and incubated in air or in 5% CO_2_ at 37°C for three days. Total RNA was extracted and used for Q-RT-PCR assays. The relative expression level of each gene in the control strain in air was set to “1”. *ACT1* served as the normalization control. Statistical significance of the differences between the values of a certain strain cultured in air and in 5% CO_2_ is indicated (*p<0.05, **p<0.01).

To elucidate the TCA-dependent CO_2_ sensing mechanism in *C*. *albicans*, we examined the transcriptional levels of key genes of the TCA cycle and Ras1-cAMP pathways. As shown in Fig 3A and 3B, the relative expression levels of the TCA cycle-related genes (*CIT1*, *ACO1*, *IDH1*, *IDH2*, *KGD1*, *KGD2*, *SDH2*, *SDH3*, and *MDH1-1*) and the cAMP signaling pathway genes (*RAS1*, *CYR1*, *TPK1*, and *TPK2*) were increased in 5% CO_2_ relative to air on YPD medium, suggesting that CO_2_ has an activating effect on both pathways. As expected, the relative expression levels of *ECE1* and *HWP1*, two hyphal-specific genes, were increased in the WT strain but not in the *cit1/cit1*, *aco1/aco1*, *sdh3/sdh3*, and *mdh1-1/mdh1-1* mutants in response to 5% CO_2_ relative to that in air (Fig 3C and 3D).

### Disruption of the TCA cycle-related genes results in a decrease in intracellular ATP and GTP-Ras1 levels

**Fig 4:**
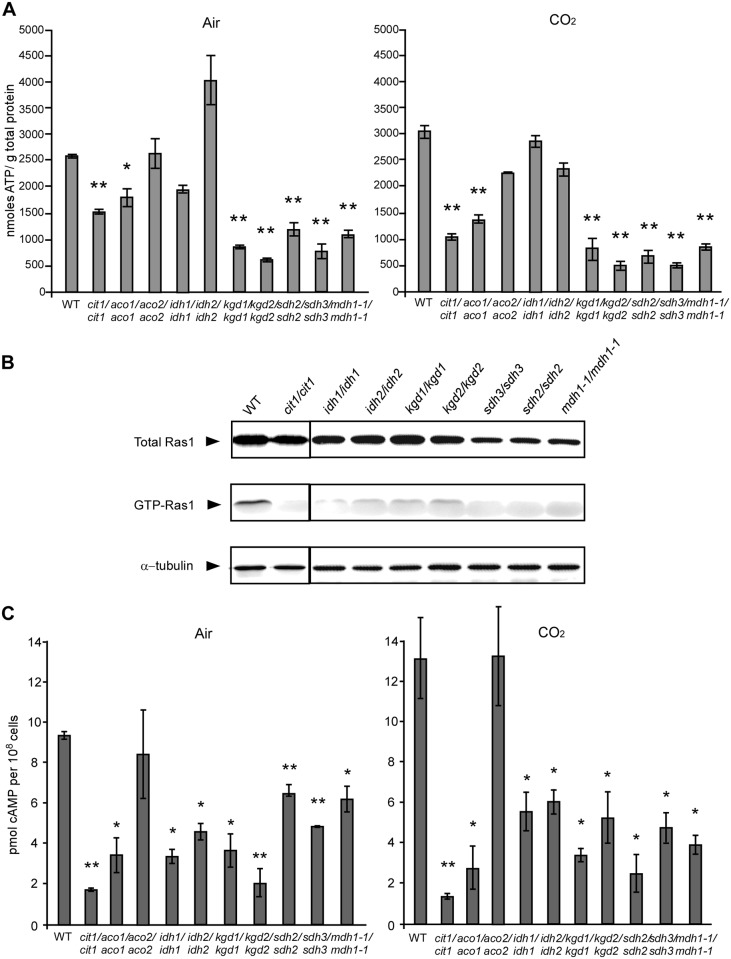
Intracellular levels of ATP (A), GTP-Ras1 (B), and cAMP (C) in the *C*. *albicans* TCA cycle mutants. Cells of the control strain (WT, SN152+) and TCA cycle mutants were grown on YPD medium plates for 16 h at 37°C in air or in 5% CO_2_. (A) Intracellular ATP levels of the WT and mutants in air or in 5% CO_2_. (B) Western blot analysis of total Ras1 and GTP-Ras1 protein levels of the WT and mutants in air. α-Tubulin served as the loading control. Two independent experiments were performed. (C) Intracellular cAMP levels of the WT and mutants in air or in 5% CO_2_. For A and C, statistical significance of the differences between the values of a certain strain and the WT is indicated (*p<0.05, **p<0.01, Student’s *t*-test, two tailed).

The cellular energy state regulates many biological processes including morphologic transitions through the Ras1-cAMP pathway in *C*. *albicans* [29] [17]. The TCA cycle is a central metabolic pathway that produces the reducing factors, NADH and FADH_2_, for the subsequent production of ATP through the ETC. We, therefore, examined intracellular ATP levels in the WT and TCA cycle mutants. As demonstrated in Fig 4A, the TCA cycle mutants (such as *kgd1/kgd1*, *kgd2/kgd2*, *sdh2/sdh2*, and *sdh3/sdh3*), which had decreased hyphal growth abilities under several culture conditions, also showed relatively low levels of intracellular ATP compared to the WT. However, the *idh2/idh2* mutant, which showed a slightly reduced hyphal growth, had a relatively high level of intracellular ATP in air. The intracellular ATP level is correlated with the activation of Ras1 in *C*. *albicans* [29]; the GTP-bound Ras1 protein represents an activated form. Western blot and IP assays demonstrated that the level of GTP-Ras1 is notably lower in the TCA cycle mutants compared to that of the WT control (Fig 4B). Consistently, a reduction of intracellular cAMP levels was observed in the corresponding TCA cycle mutants, including c*it1/cit1*, *idh1/idh1*, *idh2/idh2*, *kgd1/kgd1*, *kgd2/kgd2*, *sdh2/sdh2*, *sdh3/sdh3*, and *mdh1-1/mdh1-1* (Fig 4C).

### The TCA cycle is required for Ras1-cAMP pathway-activated hyphal growth

To further characterize the regulatory relationship between the Ras1-cAMP pathway and the TCA cycle, we generated a set of Ras1-cAMP pathway-constitutively active strains by overexpressing the activating form of *RAS1* (*RAS1V13*) or by deleting the high-affinity cyclic nucleotide phosphodiesterase-encoding gene, *PDE2*, in the TCA cycle mutants. We successfully obtained the RAS1V13-overexpressing strains in the *kgd1/kgd1*, *kgd2/kgd2*, and *sdh2/sdh2* mutants and successfully deleted *PDE2* in five mutants (*idh1/idh1*, *idh2/idh2*, *sdh2/sdh2*, *sdh3/sdh3*, and *mdh1-1/mdh1-1*). However, we were unable to overexpress *RAS1V13* or to delete *PDE2* in the remaining TCA mutants. It is possible that the constitutive activation of the Ras1-cAMP pathway may be lethal in these mutants. As shown in S7A and S8A Figs, overexpression of *RAS1V13* in the *kgd1/kgd1*, *kgd2/kgd2*, and *sdh2/sdh2* mutants had no notable effects on hyphal growth in air or in 5% CO_2_. Deletion of *PDE2* in the *idh1/idh1* and *idh2/idh2* mutants triggered hyphal growth to similar levels to that of the WT strain (S7B Fig), whereas deletion of *PDE2* in the *sdh2/sdh2*, *sdh3/sdh3*, and *mdh1-1/mdh1-1* mutants had no obvious effect on the induction of hyphal growth in air or in 5% CO_2_ (S7B and S8B Figs). These results demonstrate that some components of the TCA cycle are required for Ras1-cAMP pathway-mediated hyphal growth in *C*. *albicans*.

### The Ras1-cAMP pathway regulates the TCA cycle and CO_2_ sensing in *C*. *albicans*

**Fig 5:**
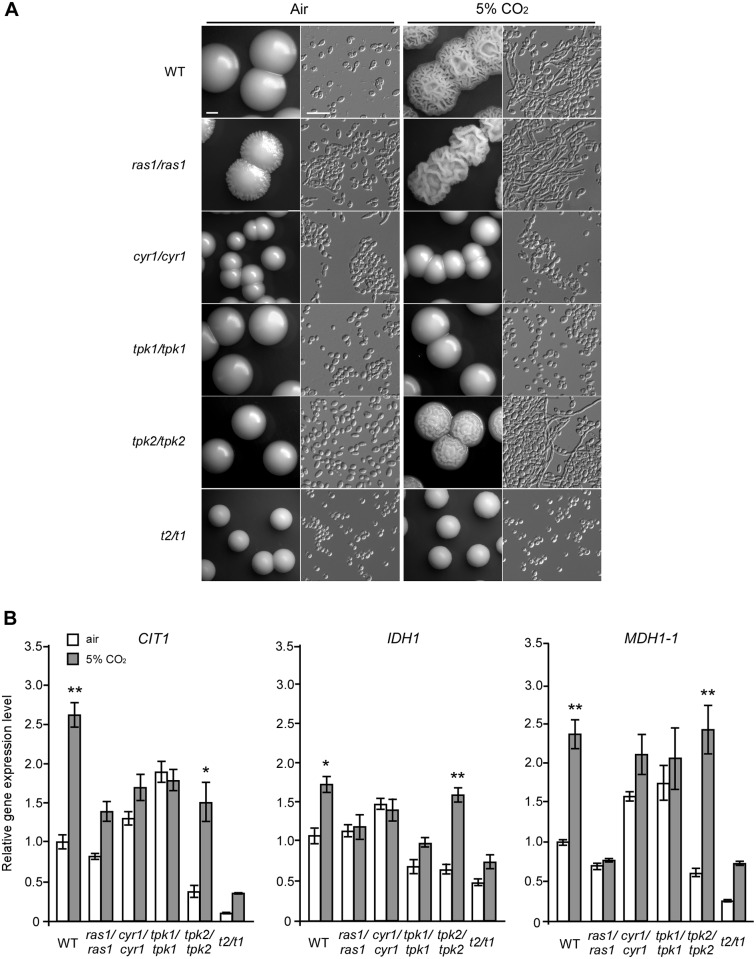
The Ras1-cAMP signaling pathway regulates CO_2_-induced hyphal growth and the expression of TCA cycle genes. (A) Colony and cellular morphological phenotypes of the Ras1-cAMP signaling pathway mutants grown on YPD at 37°C for three days in air or in 5% CO_2_. Scale bars: 1 mm for colony images; 20 μm for cellular images. (B) Relative transcriptional levels of *CIT1*, *IDH1*, and *MDH1-1* in the cAMP signaling pathway mutants. Cells of the different mutants were plated onto YPD medium plates and incubated at 37°C in air or in 5% CO_2_ for 24 h. Total RNA was then isolated and used for Q-RT-PCR assays. *t2/t1* is the *tpk2/tpk2 tpk1/tpk1* double mutant. The control strain (WT) is SN152+. Statistical significance of the differences between the values of a certain strain cultured in air and in 5% CO_2_ is indicated (*p<0.05, **p<0.01, Student’s *t*-test, two tailed).

Taking advantage of a set of homozygous deletion mutants recently generated in our lab [22], we evaluated the role of the Ras1-cAMP pathway in CO_2_-induced hyphal growth on YPD medium. As shown in Fig 5A, the *cyr1/cyr1*, *tpk1/tpk1*, and double *tpk2/tpk2 tpk1/tpk1 (t2t1)* mutants failed to undergo hyphal growth under both conditions, while the *ras1/ras1* and *tpk2/tpk2* mutants behaved as the WT control. These results suggest that the adenylyl cyclase, Cyr1, and the PKA catalytic subunit, Tpk1, are required for CO_2_-induced hyphal growth under this culture condition in *C*. *albicans*. Given the similar phenotypes of the TCA cycle and the Ras1-cAMP pathway mutants in response to elevated CO_2_ levels, we next examined the transcriptional levels of three representative genes of the TCA cycle, *CIT1*, *IDH1*, and *MDH1-1*, in the WT, *ras1/ras1*, *cyr1/cyr1*, *tpk1/tpk1*, *tpk2/tpk2*, and double *tpk2/tpk2 tpk1/tpk1* mutants by Q-RT-PCR. As shown in Fig 5B, deletion of *TPK2* had no significant effects on the CO_2_-induced expression of *CIT1*, *IDH1*, and *MDH1-1*, whereas deletion of *RAS1*, *CYR1*, *TPK1*, or both *TPK* genes severely reduced (and in some cases completely abolished) the CO_2_-induced expression of *CIT1*, *IDH1*, and *MDH1-1*. These results suggest that Tpk1, but not Tpk2, of the Ras1-cAMP pathway, plays a major role in the regulation of the TCA cycle and CO_2_ sensing in *C*. *albicans*. The TCA cycle and Ras1-cAMP pathways inter-regulate each other and may control hyphal growth and CO_2_ sensing in a coordinate manner.

### Transcription factors Efg1 and Sfl2 control the TCA cycle and CO_2_-induced hyphal growth

**Fig 6:**
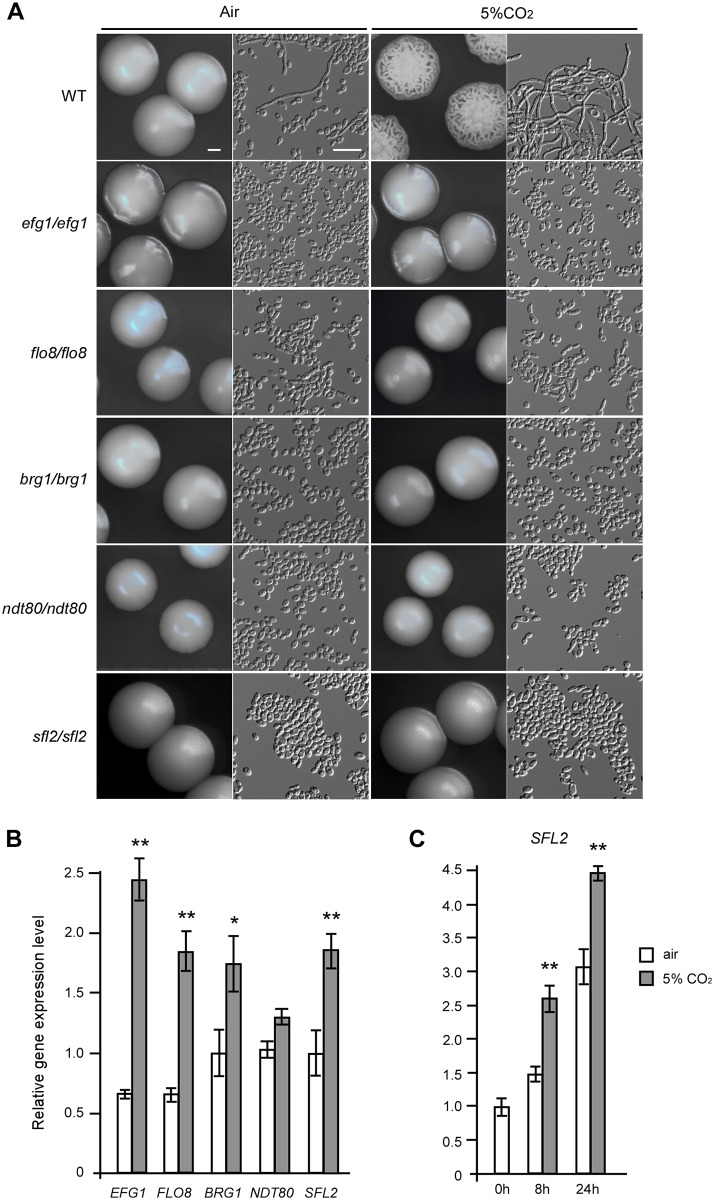
Transcription factors required for CO_2_-induced hyphal growth. The control strain (WT) is SN152+. For A and B, cells were grown on YPD medium plates at 37°C in air or in 5% CO_2_ for three days. (A) Colony and cellular morphologies of the *efg1/efg1*, *flo8/flo8*, *brg1/brg1*, *ndt80/ndt80*, and *sfl2/sfl2* mutants. Scale bars: 1 mm for colony images; 20 μm for cellular images. (B) Relative expression levels of *EFG1*, *FLO8*, *BRG1*, *NDT80*, and *SFL2* in air or in 5% CO_2_ in WT cells. (C) Relative expression levels of *SFL2* in air or in 5% CO_2_ after 0, 8, and 24 h of growth on Lee’s glucose medium at 30°C in air or in 5% CO_2_ in WT cells. Statistical significance of the differences between the values of a certain strain cultured in air and in 5% CO_2_ is indicated (*p<0.05, **p<0.01, Student’s *t*-test, two tailed).

**Fig 7:**
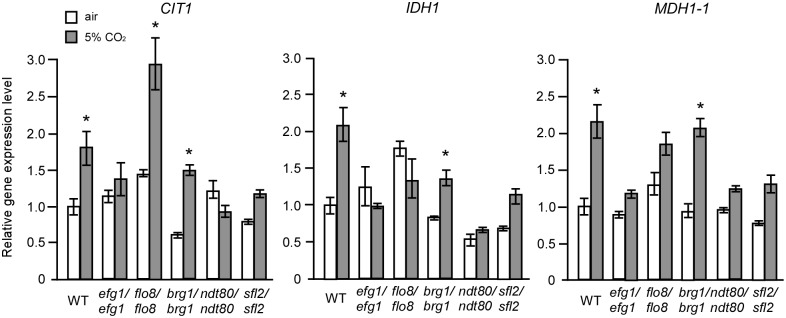
Relative expression levels of *CIT1*, *IDH1*, and *MDH1-1* in the hyphal growth-related transcription factor mutants in air or in 5% CO_2_. The control strain (WT) is SN152+. Cells were grown on YPD medium plates at 37°C in air or in 5% CO_2_ for 24 h. Total RNA was isolated for Q-RT-PCR assays. “*”, indicates significant differences between the values in air and in 5% CO_2_ (p<0.05, *Student*’s *t*-test, two tailed).

**Fig 8:**
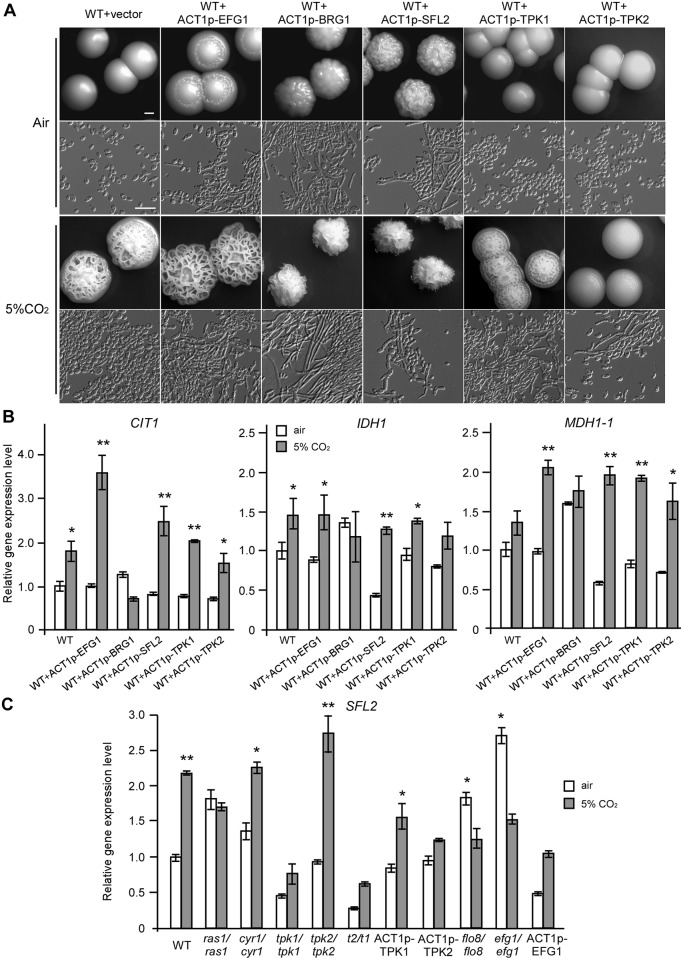
Effects of overexpressing *EFG1*, *BRG1*, *SFL2*, *TPK1*, and *TPK2* on CO_2_-induced hyphal growth and expression of TCA cycle-related genes. Cells were cultured on YPD medium plates at 37°C in air or in 5% CO_2_ for three days. (A) Colony and cellular morphologies of overexpression strains. Scale bars: 1mm for colony images; 20μm for cellular images. (B) Relative expression levels of TCA cycle-related genes in the overexpression strains. The WT+vector strain (CAI4+pACT1) served as a control. (C) Relative expression levels of*SFL2* in the Ras1-cAMP signaling pathway mutants and overexpression strains. Cells were grown on YPD medium plates at 37°C in air or in 5%CO_2_ for 24h. The relative expression level of the control strain (SN152+) in air was set to “1”. *p<0.05, **p<0.01, *Student*’s *t*-test, two tailed.

To reveal the transcriptional regulatory mechanisms of the TCA cycle and CO_2_-induced hyphal growth, we examined approximately 30 homozygous transcription factor deletion mutants for morphological changes in air and in 5% CO_2_. As shown in Fig 6A, we identified five transcription factor mutants (*brg1/brg1*, *efg1/efg1*, *flo8/flo8*, *ndt80/ndt80*, and *sfl2/sfl2*) that were unable to undergo filamentous growth in 5% CO_2_. Q-RT-PCR assays demonstrated that the transcriptional levels of *BRG1*, *EFG1*, *FLO8*, *SFL2*, but not *NDT80*, were significantly increased under the high CO_2_ condition, relative to those in air (Fig 6B). The relative expression levels of *SFL2* increased in response to 5% CO_2_ after 8 or 24 hours of growth at 30°C (Fig 6C). Further experiments demonstrated that the transcriptional levels of three TCA cycle-related genes, *CIT1*, *IDH1*, and *MDH1-1*, were significantly increased in response to 5% CO_2_ in the WT and *brg1/brg1* strains, but not in the *ndt80/ndt80*, *efg1/efg1*, and *sfl2/sfl2* mutants (Fig 7). *CIT1* was also significantly increased in 5% CO_2_ in the *flo8/flo8* mutant. Compared to the WT strain, the strains overexpressing *EFG1* and *SFL2* showed more robust hyphal growth and increased expression of *CIT1* and *MDH1-1* in 5% CO_2_, whereas overexpression of *BRG1* promoted hyphal growth, but did not induce the expression of the TCA cycle-related genes (Fig 8A and 8B). Actually, overexpression of *BRG1* had a suppressing effect on the transcription of *CIT1*, *IDH1*, and *MDH1-1*(Fig 8B). These results indicate that Efg1 and Sfl2 play major roles in the transcriptional regulation of TCA cycle and CO_2_-mediated hyphal growth. Overexpression of *TPK1* increased the transcriptional expression of *MDH1-1* (Fig 8B) and had a notable stimulating effect on hyphal growth in the presence of 5% CO_2_ (Fig 8A). These results confirm that Tpk1 plays major roles in the regulation of the TCA cycle and CO_2_ sensing, possibly through the transcription factors Efg1 and Sfl2.

### Tpk1 and Efg1 are required for CO_2_-induced *SFL2* expression

The adenylyl cyclase Cyr1 is an important sensor of CO_2_ and Efg1 is a downstream transcription factor of the Ras1-cAMP pathway [25, 42]. It is, therefore, reasonable that Efg1 may play key roles in TCA cycle- and CO_2_-mediated hyphal growth in *C*. *albicans*. To establish a link between the Ras1-cAMP pathway and the Sfl2 transcription factor, we performed Q-RT-PCR assays on a set of overexpressing and deletion strains of this pathway. As demonstrated in Fig 8C, the expression of *SFL2* was induced in the WT, *cyr1/cyr1*, and *tpk2/tpk2* strains but not in the *ras1/ras1*, *tpk1/tpk1*, *tpk2/tpk2 tpk1/tpk1 (t2t1)*, and *efg1/efg1* mutants in response to 5% CO_2_. These results suggest that the Ras1-cAMP pathway directly or indirectly regulates CO_2_-induced *SFL2* expression and that Tpk1 and Efg1 play major roles in this regulation.

### Sfl2 is required for elevated CO_2_ level-altered gene expression

**Fig 9:**
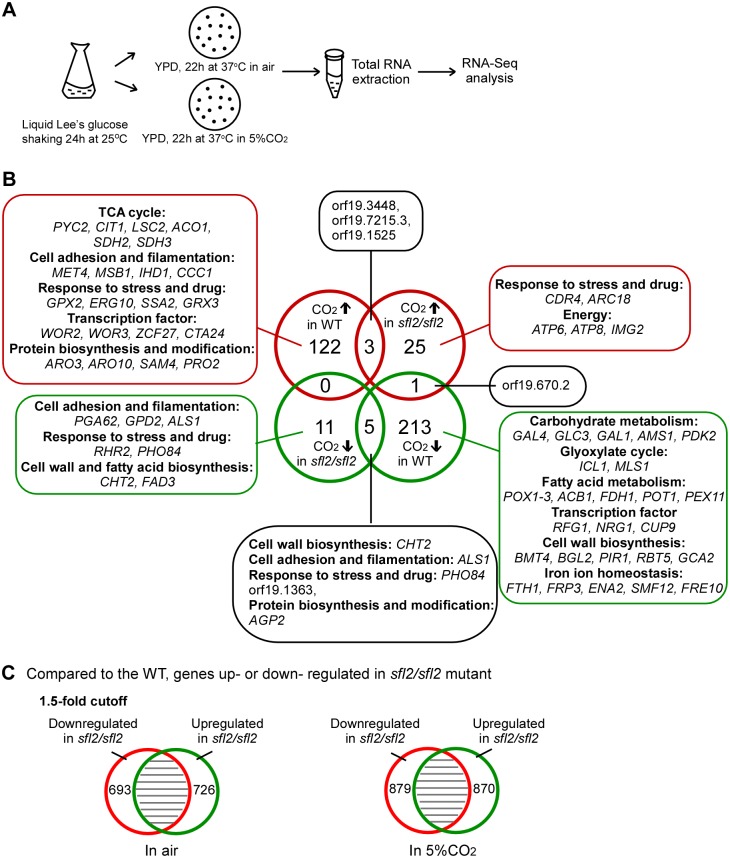
A comparison of global expression profiles of the WT and *sfl2/sfl2* mutant in air and 5% CO_2_. (A) Schematic diagram of experimental procedures for RNA-Seq analysis. (B) Overlap between differentially expressed genes of the WT and *sfl2/sfl2* mutant in air and 5% CO_2_. Red circles, CO_2_-upregulated genes in the WT (left, 122) and *sfl2/sfl2* mutant (right, 25); green circles, CO_2_-downregulated genes in the WT (right, 213) and *sfl2/sfl2* mutant (left, 11). Eight genes (three upregulated and five downregulated) were found to be altered both in the WT and *sfl2/sfl2* mutant by the elevated level of CO_2_. (C) Compared to the WT, genes upregulated or downregulated in the *sfl2/sfl2* mutant in air and 5% CO_2_. A 1.5-fold cut-off was used for B and C. Detailed analyses of the RNA-Seq data are presented in S1 Dataset.

We next performed RNA-Seq analysis to elucidate the function of Sfl2 in CO_2_ sensing in *C*. *albicans*. As mentioned earlier, CO_2_ is a potent inducer of hyphal formation [42]. To identify CO_2_-regulated genes in the early phase of hyphal induction, prior to cell fate commitment to the hyphal form, we incubated *C*. *albicans* cells of the WT and *sfl2/sfl2* mutants on YPD medium in air or 5% CO_2_ for 22 hours at 37°C (Fig 9A). This time point was chosen since cells at this stage are in early exponential growth and are not yet beginning to form hyphae. Therefore, most genes identified here should be CO_2_-responsive, and gene alterations resulting from hyphal growth should be trivial.

As shown in Fig 9B, there were 122 CO_2_-upregulated and 213 CO_2_-downregulated genes in the WT, whereas there were only 25 CO_2_-upregulated and 11 CO_2_-downregulated genes in the *sfl2/sfl2* mutant (using a 1.5-fold cut-off). This remarkable difference in the number of differentially expressed genes between the WT and *sfl2/sfl2* mutant strains suggests that Sfl2 plays a fundamental role in the transcriptional control of CO_2_-regulated genes.

CO_2_-upregulated genes in the WT include the following (using a 1.5-fold cut-off): (1) Genes related to the TCA cycle (e.g., *CIT1*, *ACO1*, *ACO2*, *SDH2*, and *SDH3*). This result is consistent with our Q-RT-PCR analysis in which *C*. *albicans* cells were incubated in 5% CO_2_ for an extended period of time (three days, Fig 3). (2) Genes involved in cell adhesion and filamentation (e.g., orf19.5126, *MET4*, *IHD1*, *MSB1*, *MSB2*, and *CCC1*). (3) Genes responsive to stress and drugs (e.g., *GPX2*, *ERG10*, *SSA2*, *GRX3*, and *AHA1*). (4) Amino acid synthesis-related genes (e.g., *ARO10*, *ARO3*, *SAM4*, *PRO2*, and *HIS7*). *ARO* genes are associated with the ehrlich pathway and aromatic amino acid synthesis; the TCA cycle may regulate these processes through its intermediate metabolites. (5) Genes encoding transcription factors (e.g., *WOR2*, *WOR3*, *ZCF27*, *TEC1*, and *EFG1*). Wor2, Wor3, and Efg1 are key regulators of white-opaque switching in *C*. *albicans*. Tec1 and Efg1 are well known for their involvement in the regulation of hyphal growth [30].

We also identified a set of CO_2_-downregulated genes in the WT. Carbonic anhydrase catalyzes the hydration reaction of CO_2_. As expected, the carbonic anhydrase-encoding gene, *NCE103*, was downregulated in 5% CO_2_. A number of metabolism-related genes were downregulated in 5% CO_2_, for example, the glyoxylate cycle-specific genes (*MLS1* and *ICL1*), fermentative metabolism-related genes of sugars (*HGT1*, *HGT8*, *HGT19*, *PCK1*, *ADH5*, *IFE1*, and *IFE2*), and fatty acid metabolism-related genes (*POX1-3*, *ACB1*, *FDH1*, *POT1*, and *PEX11*). Consistent with the filament-inducing role of CO_2_, several transcriptional repressor genes (*CUP9*, *NRG1*, and *RFG1*) were downregulated in 5% CO_2_. Many of these differentially expressed genes, especially transcription factor-encoding genes, have been reported as Sfl2-binding targets [40], suggesting that Sfl2 may function through the direct regulation of these hyphal growth regulators.

In the *sfl2/sfl2* mutant, genes of known function in response to elevated levels of CO_2_ include the following: (1) Energy and fatty acid metabolism-related genes (*ATP6*, *ATP8*, *IMG2*, and *FAD3*) and two stress and drug response genes (*CDR4* and *ARC18*) were upregulated. (2) Interestingly, cell wall and hyphal growth-related genes (*PGA62*, *GPD2*, *ALS1*, and *CHT2*) were downregulated. The expression of these genes may be independent of the regulation of Sfl2 in *C*. *albicans*.

### Differentially expressed genes between the WT and *sfl2/sfl2* mutant

We next compared the differentially expressed genes between the WT and *sfl2/sfl2* mutant (Fig 9B and 9C). Using a 1.5-fold cut-off, there are 1,419 and 1,749 genes differentially expressed between the WT and *sfl2/sfl2* mutant in air and in 5% CO_2_, respectively. However, when a 2-fold cut-off is used, the numbers are dramatically decreased to 400 and 607, in air and in 5% CO_2_, respectively (S1 Dataset). We note that in general, there are about 30% more differentially expressed genes in 5% CO_2_ than in air. This difference is likely due to the different responses between the WT and *sfl2/sfl2* mutant to elevated CO_2_ levels. Indeed, the elevated CO_2_-levels had a much weaker effect on the global gene expression profile of the *sfl2/sfl2* mutant compared to WT (Fig 9B).

### Sfl2 binds to the promoters of TCA cycle-related and transcriptional regulator-encoding genes under high CO_2_ levels

**Fig 10:**
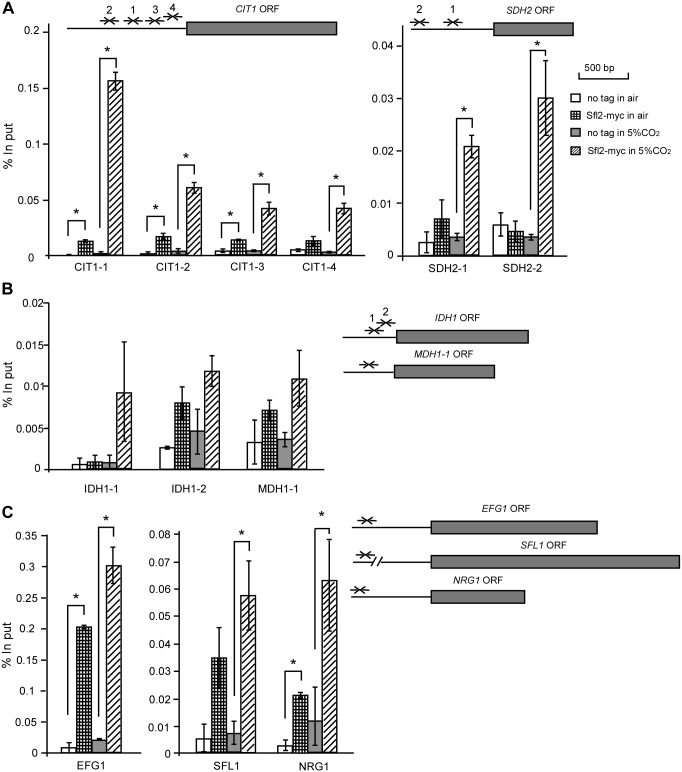
Sfl2 binds to the promoters of TCA cycle genes (A and B) and specific transcription factors (C) in 5% CO_2_. Chromatin immunoprecipitation (ChIP) assays were performed in a Sfl2-Myc-tagged strain grown on YPD medium in air or in 5% CO_2_. An untagged strain served as a control. Error bars represent standard errors of two independent experiments. The localization of primer pairs used for quantitative PCR is indicated in the promoter regions. The relative Sfl2-binding ability is indicated by the percentage of input DNA. *p<0.05, *Student*’s *t*-test, two tailed.

To further characterize the regulatory mechanisms of Sfl2, we performed ChIP-qPCR assays to identify target genes directly regulated by Sfl2. The promoters of all TCA cycle genes were examined. We found that Sfl2 specifically bound to the promoters of *CIT1* and *SDH2* in 5% CO_2_ but not in air (Fig 10A). The enrichment levels for Sfl2 binding in the promoters of *IDH1* and *MDH1-1* were weak both in air and in 5% CO_2_ (Fig 10B). We further found that Sfl2 bound to the promoters of *EFG1*, *SFL1*, and *NRG1* both in air and in 5% CO_2_ (Fig 10C). Overall, the enrichment for binding by Sfl2 in 5% CO_2_ was higher than that in air. We also found some potential Sfl2-binding sites in the promoters of *CIT1*,*SDH2*, *IDH1*, *MDH1-1*, *EFG1*, *NRG1*, and *SFL1* according to the consensus sequence (AATAGAA) identified by Znaidi *et al*. (2013) [40]. Our results suggest that Sfl2 not only directly binds to the promoters of TCA cycle-related genes, but also indirectly regulates the TCA cycle and CO_2_-induced hyphal development by binding to the promoters of other transcription factors.

## Discussion

*C*. *albicans* is well adapted to its mammalian host and, unlike most fungi, is not known to exist in the external environment [8, 44]. The abilities of *C*. *albicans* to utilize host-derived nutrients and to rapidly adapt to the ever-changing host environment are critical for its pathogenic and commensal life styles [45]. Unlike *S*. *cerevisiae*, *C*. *albicans* is a Crabtree-negative species that exclusively uses respiration to produce energy under aerobic conditions [46]. This metabolic feature may be crucial for *C*. *albicans* to efficiently use host-derived nutrients. To uncover the adaptive mechanisms of *C*. *albicans* in the context of metabolism, we performed a comprehensive study of the TCA cycle, the central pathway of metabolism, using a series of TCA pathway mutants. Via integration with the Ras1-cAMP pathway and transcriptional regulation, the TCA cycle plays critical roles in the regulation of carbon source utilization, hyphal growth, and CO_2_ sensing in *C*. *albicans*.

### The TCA cycle regulates carbon source utilization

The TCA cycle is the second stage of respiration after glycolysis. It produces ATP and the electron carriers, NADH and FADH_2_, for the ETC. We found that disruption of key proteins of the TCA cycle (e.g., Cit1, Aco1, Kgd1, Kgd2, or Mdh1-1) led to severe growth defects on non-fermentative carbon sources (S1, S2 and S3 Figs), which can only be metabolized to produce energy through respiration. Some metabolic enzymes of the TCA cycle are encoded by two or three genes. For example, *IDH1* and *IDH2* encode two subunits of isocitrate dehydrogenase, and *SDH2*, *SDH3*, and *SDH4* encode three succinate dehydrogenase subunits (Fig 1). The *idh1/idh1* and *idh2/idh2* mutants displayed similar growth rates to the WT strain in all tested media. However, both the iron-sulfur subunit of succinate dehydrogenase, Sdh2, and the flavoprotein subunit, Sdh3, are required for efficient utilization of non-fermentative carbon sources, whereas Sdh4 does not appear to play a role in this regulation (S1, S2 and S3 Figs). Of note, the SDH complex is also a component of the ETC. These results suggest that different subunits or isoforms of TCA cycle enzymes may play redundant and distinct roles in *C*. *albicans*. Deletion of *CIT1* and *ACO1* genes, encoding citrate synthase and aconitase enzymes, respectively, resulted in major growth defects even in media containing fermentative sugars, such as glucose and sucrose (S1 Fig). This growth defect could be due to the accumulation of toxic intermediate metabolites, such as acetic acid. Our results suggest that the TCA cycle regulates carbon source utilization and energy metabolism in *C*. *albicans*. Consistent with a previous study, deletion of *MCU1*, which encodes a mitochondrial protein required for the function of the TCA cycle, also caused severe defects in carbon source utilization and hyphal growth in *C*. *albicans* [47].

In minimal media, most *C*. *albicans* TCA cycle mutants exhibited similar growth phenotypes to their orthologous mutants in *S*. *cerevisiae*, indicating the conserved features of the TCA cycle. However, the *idh1/idh1*, *idh2/idh2*, *sdh4/shd4*, *fum11/fum11*, and *fum12/fum12* mutants of *C*. *albicans* were able to grow on amino acid or non-fermentable carbon source media (minimal media, S1 Fig), whereas the *idh1*, *idh2*, *sdh4*, and *fum1* mutants of *S*. *cerevisiae* exhibited severe growth defects under similar culture conditions [48, 49]. These results imply that, as a commensal of humans, *C*. *albicans* is better equipped at utilizing diverse carbon sources than *S*. *cerevisiae*.

Moreover, given its central position in cellular metabolism, including its roles in lipid and amino acid synthesis, the TCA cycle may also regulate hyphal development through other metabolic intermediates and pathways. For example, the enzymes citrate synthase, aconitase, and isocitrate dehydrogenase are responsible for α-ketoglutarate synthesis, which is required for the production of glutamate, a precursor for the amino acids arginine, proline, and glutamic acid. Indeed, mutants lacking aconitase in *S*. *cerevisiae* exhibit growth defects in minimal media lacking glutamate [50]. Consistently, we observed that the *cit1/cit1* and *aco1/aco1* mutants of *C*. *albicans* were able to grow on rich medium (YPD) but were unable to grow on minimal medium (Lee’s glucose and YNB, Fig 2, S1 Fig and S1 Table).

### The TCA and Ras1-cAMP pathways coordinately regulate hyphal development in response to elevated CO_2_ levels

The Ras1-cAMP pathway is the central regulator of hyphal growth in *C*. *albicans* [17, 51]. The adenylyl cyclase Cyr1 catalyzes the conversion of ATP to the second messenger cAMP. Grahl *et al*. (2015) demonstrated that the ATP pool serves as a “checkpoint” in the activation of Ras signaling under filament-inducing conditions [29]. When the intracellular ATP level is low, the activated form, GTP-Ras1, turns over to the inactivated form, GDP-Ras1, in a Cyr1-Ira2 dependent manner. The mitochondrial respiratory chain provides a major source of ATP for the cell. Mutants defective in the ETC are unable to establish a high intracellular ATP level and thus fail to undergo hyphal growth [29]. Consistently, here we showed that disruption of the TCA cycle leads to reduced intracellular ATP levels and defects in hyphal growth. This is not surprising since the TCA cycle generates NADH and FADH_2_, which are required for ATP production during subsequent ETC-mediated oxidation events. The disruption of TCA cycle-related genes had notable effects on intracellular ATP levels and Ras GTP-binding (Fig 4). Decreased GTP-Ras1 levels are directly related to the inactivation of cAMP signaling and hyphal growth in *C*. *albicans*. The TCA cycle is required for utilizing non-fermentable carbon sources to generate energy. We observed that there is a correlation between the ability to undergo hyphal growth and the ability to utilize non-fermentable carbon sources (Fig 1, S1, S2 and S3 Figs, Table 1 and S1 Table). Constitutive activation of the Ras1-cAMP signaling pathway by overexpressing *RAS1V13* or by deleting *PDE2* in TCA cycle mutants induced hyphal growth in the *idh1/idh1* and *idh2/idh2* mutants, but not in other TCA cycle mutants in air or in 5% CO_2_. These results suggest that the TCA cycle regulates CO_2_ sensing and hyphal growth in *C*. *albicans* through modulation of intracellular ATP levels and through the activation of Ras1 signaling. Artificially activated-Ras1-cAMP signaling could not suppress the hyphal growth defect of the TCA cycle mutants, suggesting that this cycle is required for basal levels of hyphal development in *C*. *albicans*. An alternative possibility is that the TCA cycle and Ras1/cAMP signaling pathways may function independently on hyphal growth.

### CO_2_ functions as a link between the TCA cycle and Ras1-cAMP pathways

Although disruption of the TCA cycle blocked or attenuated hyphal growth on Lee’s glucose medium at 37°C and Lee’s GlcNAc medium at 30°C, the TCA cycle mutants were still able to form hyphae on Lee’s GlcNAc, YPD + serum and Spider media at 37°C (Table 1 and S4 Fig), suggesting that the observed hyphal growth defects are condition-dependent. CO_2_ is an end product of respiratory metabolism as well as a potent inducer of hyphal growth in *C*. *albicans* [42]. CO_2_ functions through the activation of adenylyl cyclase and through an unknown pathway [31]. Here we demonstrate that some components of the TCA cycle are required for CO_2_-induced hyphal growth in *C*. *albicans* (Table 1 and Fig 2 and S5 Fig). CO_2_ promotes the transcriptional expression of TCA cycle genes possibly through the activation of the cAMP signaling pathway (Figs 3, 4 and 5). The adenylyl cyclase Cyr1 and catalytic subunit isoform Tpk1, but not Tpk2, play critical roles in CO_2_-induced hyphal growth and expression of TCA cycle genes (Fig 5). In the model yeast *S*. *cerevisiae*, high levels of CO_2_ can also induce the transcription of respiratory metabolism-related genes and can cause an increase in intracellular ATP demand [52, 53], suggesting that the regulatory mechanisms of CO_2_-induced gene expression are conserved. This metabolic response to increased CO_2_ levels may benefit *C*. *albicans* as both a commensal and a pathogen. Given the relative levels of oxygen (1% or lower) and high levels of CO_2_ (4.5–30%) in host niches (e.g., the lower gastrointestinal tract), elevated CO_2_ levels would facilitate respiratory metabolism in this Crabtree-negative species, which may be an adaptive mechanism for existing as a commensal or pathogen in the host that depends on environmental cues. As mentioned earlier, the Ras1-cAMP pathway regulates the TCA cycle and mitochondrial metabolic activity in *C*. *albicans*. CO_2_ produced by respiratory metabolism could function as an intracellular and intercellular signal to activate the Ras1-cAMP pathway. Indeed, it has been demonstrated that CO_2_ can act as an intercellular signal for cell-cell communication and self-induced hyphal growth in *C*. *albicans* [43]. Therefore, we propose that CO_2_ functions to link the TCA cycle and Ras1-cAMP pathways.

### Transcriptional regulation of the TCA cycle and CO_2_-induced hyphal growth

We identified Sfl2 and Efg1 as two major transcriptional regulators of CO_2_-induced hyphal growth and the TCA cycle in *C*. *albicans* (Figs 6, 7 and 8). Efg1 is a downstream transcription factor of the Ras1-cAMP pathway and plays a global role in transcriptional regulation in a number of important developmental processes in *C*. *albicans* [24–26, 54]. Deletion of *EFG1* also has fundamental consequences on metabolism, likely due to perturbations of this global transcriptional profile. Moreover, Efg1 and Ace2 are also involved in the regulation of metabolism in *C*. *albicans* through Bcr1 and Brg1 [55]. It has been showed that Efg1 induces the expression of glycolytic genes and represses the expression of oxidative genes [56]. We have reported that Flo8 plays a critical role in CO_2_-induced hyphal growth and white-opaque switching in *C*. *albicans* [31]. In this study, we also found that deletion of *FLO8* or *BRG1*, encoding a GATA-type transcription factor, resulted in severe defects in hyphal growth in response to CO_2_ (Fig 6). However, deletion of *BRG1* had no obvious effects on the transcriptional expression of TCA cycle genes (Fig 7). Since elevated CO_2_ levels promote the expression of *SFL2*, which encodes a heat shock factor-type transcription factor [38, 39], we chose to explore the mechanisms of this regulation. Consistently, deletion of *SFL2* led to severe hyphal growth defects and the failure to induce TCA cycle gene expression in high CO_2_ conditions (Figs 6 and 7). ChIP assays under these conditions demonstrated that Sfl2 bound to the promoters of *CIT1* and *SDH2* (Fig 10). RNA-Seq analysis further demonstrated that Sfl2 is essential for CO_2_-altered gene expression at the global transcriptome level. These results suggest that Sfl2 plays critical roles in CO_2_-induced responses in *C*. *albicans*.

Transcriptional analysis demonstrated that the Ras1-cAMP pathway and Efg1 play important roles in the control of *SFL2* expression in response to high CO_2_ levels (Fig 8). Our results indicate that the Tpk1 catalytic subunit, but not the Tpk2 subunit, plays a major role in this regulation. Znaidi et al. (2013) have shown that Sfl2 directly controls the expression of a series of positive hyphal growth regulators, such as *UME6* and *TEC1*, as well as negative hyphal growth regulators, such as *NRG1* and *RFG1*, in *C*. *albicans* [40]. Sfl2 also physically interacts with Efg1 [40], a major transcriptional regulator of morphogenesis. The expression of *SFL2* was significantly increased in the *efg1/efg1* mutant in air (Fig 8C), suggesting that Efg1 regulates *SFL2* at the transcriptional level. We found that Sfl2 is highly enriched at the promoters of *SFL1*, *EFG1* and *NRG1* and several TCA-related genes (Fig 10). Consistent with these results, previous studies found that deletion of *SFL2* blocked CO_2_–induced hyphal growth [38] and overexpression of *SFL2* promoted hyphal growth in a Flo8- and Efg1-dependent manner [39]. Interestingly, the *SFL2* orthologue in *C*. *dubliniensis*, a species closely related to *C*. *albicans*, is highly divergent from *CaSFL2* [38]. This divergence may account for the lack of observed CO_2_ responses in *C*. *dubliniensis*. In summary, the transcription factor Sfl2 plays a central role in the transcriptional control of global gene expression and hyphal growth in response to elevated CO_2_ levels. In addition, the TCA cycle, integrated with the Ras1-cAMP signaling pathway and specific transcription factors, is also important for CO_2_ sensing in *C*. *albicans*. Together, CO_2_ and ATP may function as molecular links between the TCA cycle and Ras1-cAMP signaling pathways.

**Fig 11:**
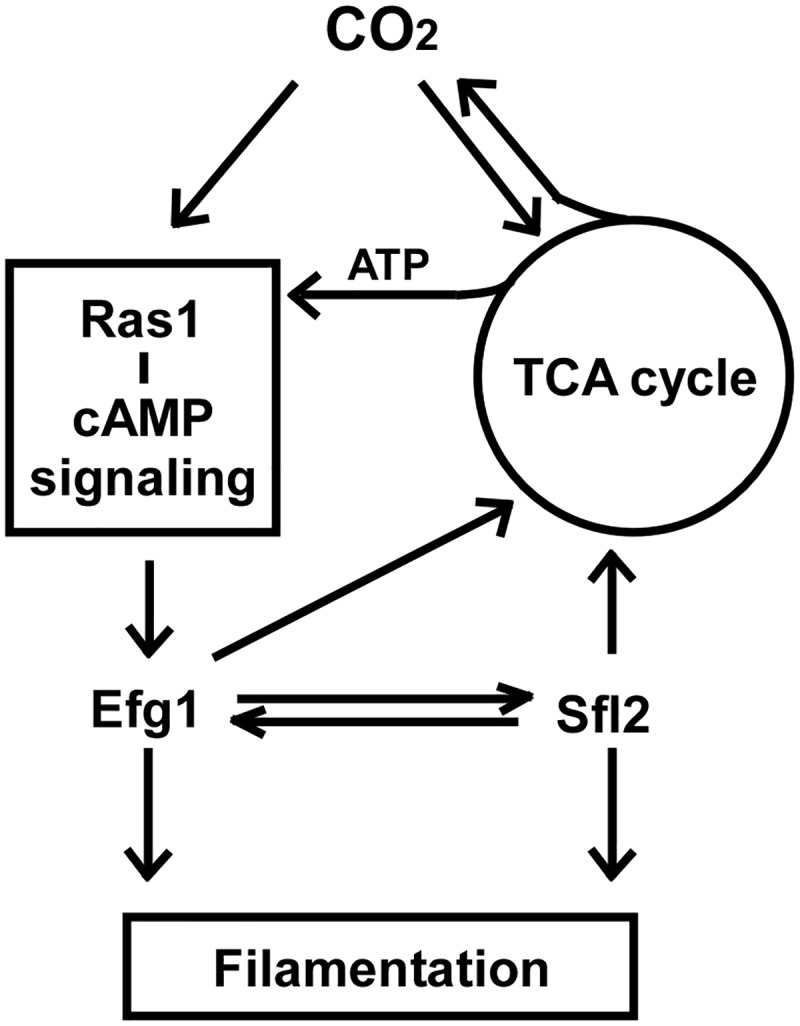
Model of the co-regulatory relationship of the TCA cycle and Ras1-cAMP pathway. Both pathways play key roles in CO_2_ sensing and CO_2_-induced hyphal growth. The Ras1-cAMP pathway controls the TCA cycle predominantly through transcriptional regulators Efg1 and Sfl2. In turn, the TCA cycle affects the Ras1-cAMP pathway by altering the intracellular levels of ATP and the signaling molecule CO_2_. Efg1 and Sfl2 likely regulate each other through both transcriptional alterations and physical interactions.

The conserved Ras1-cAMP pathway functions as an important regulator of respiratory activity in the model yeast *S*. *cerevisiae* [28, 57, 58]. Activation of this pathway increases the mitochondrial enzyme content and the transcriptional levels of genes encoding respiratory metabolism [57]. In turn, dysfunctional mitochondria modulate the Ras1-cAMP signaling pathway and affect morphological transitions in *S*. *cerevisiae* [58]. In this study, we demonstrate that the TCA cycle and Ras1-cAMP signaling pathways coordinately regulate filamentous growth and CO_2_ sensing in *C*. *albicans*. ATP generated from respiratory metabolism is required for the synthesis of the second messenger cAMP and for formation of GTP-Ras1. Environmental CO_2_ or CO_2_-derived from the TCA cycle activates the Ras1-cAMP signaling pathway, which regulates the TCA cycle through the transcription factors, Efg1 and Sfl2. These two transcription factors also control hyphal growth in *C*. *albicans*. Our results suggest that in *C*. *albicans*, the TCA cycle and Ras1-cAMP signaling pathways regulate each other, and that the transcription factors Efg1 and Sfl2 and the small molecules CO_2_ and ATP function as linkers between the two pathways (Fig 11). However, under hypoxic conditions, the regulatory mechanism could be different. It has been shown that Efg1 regulates the expression of a different set of genes under hypoxic conditions compared to those of normoxic conditions [55, 56].

## Materials and methods

### Strains and growth conditions

All strains used in this study are listed in S2 Table. YPD medium (20 g/L glucose, 20 g/L peptone, 10 g/L yeast extract) was used for routine growth of *C*. *albicans* and for intracellular ATP detection and Ras1-GTP activity assays. YPD, YPD + 10% fetal bovine serum, modified Lee’s glucose, Lee’s GlcNAc [59], Spider [60], and YPD agar + fetal bovine serum media were used to assess hyphal formation. For hyphal induction assays in S4 Fig, cells initially grown on YPD medium plates at 30°C were collected, washed, and inoculated into liquid YPD + 10% serum medium or plated onto solid YPD + serum medium plates. To make YPD + serum plates, about one mL of FBS was spread on the surface of YPD agar. For all solid media cultures, cellular morphologies were examined to assess hyphal growth. All experiments were performed under normoxic conditions.

Media for spot growth assays: A) yeast nitrogen base (YNB with (NH4)_2_SO_4_) agar containing 2% GlcNAc, 2% glucose, 2% sucrose, 2% fructose, seven amino acids (1 mM of alanine or A, arginine or R, glutamine or Q, glutamic acid or E, asparagine or N, proline or P, and serine or S), 2% ethanol, or 2% glycerol. B) Lee’s solid medium containing 1.25% GlcNAc, 1.25% glucose, 1.25% mannitol, 4% glycerol, 3% ethanol plus 2% glycerol, 2% sodium citrate. Media for growth rate assays: YPD, and YNB with (NH4)_2_SO_4_ plus 2% glucose, 2% glycerol, or 2% ethanol media.

### Growth curve assays

*C*. *albicans* cells of the WT strain, TCA cycle and glyoxylate bypass gene mutants were initially grown in liquid YPD to stationary phase at 30°C, and then collected and washed with PBS twice. 6 x 10^6^ cells were inoculated into 3 mL of YPD, YNB + 2% glucose, YNB + 2% glycerol, YNB + 2% ethanol media. The cells were incubated at 37°C or 30°C with shaking at 200 rpm. Cell densities were detected at different time points. Three independent repeats were performed.

### Construction of *C*. *albicans* mutant strains

Primers used in this study are listed in S3 Table. Fusion PCR strategies [41] were used to generate the TCA cycle and glyoxylate bypass gene deletion mutants. To delete the first allele, the strain SN152 was transformed with the fusion PCR product of *CdARG4* flanked by 5’- and 3’-flanking fragments of the corresponding gene. The cells were plated onto SD-arginine medium for selective growth. The correct integration of transformants was verified by PCR using two pairs of oligonucleotides (targeting gene-CHF and ARG4-CHR; ARG4-CHF and target gene-CHR). The heterozygous strains were then used for deleting the second allele of the corresponding gene. The cells were transformed with fusion PCR products of *CdHIS1* flanked by the 5’- and 3’-fragments of corresponding genes. The transformants were selected on plates with SD medium lacking both arginine and histidine. The correct null mutants were first verified by PCR using two pairs of oligonucleotides (target gene-CHF and HIS1-CHF; target gene-CHR and HIS1-CHR for correct genomic integration checking). Another pair of oligonucleotides (target gene-orf-F and target gene-orf-R) were used to confirmed the complete deletion of the ORF regions. A similar strategy was used to delete *PCK1* and *PYC2*. *CdARG4* and *CmLEU2* were used as the selective markers. Plasmids pSN69, pSN52 or pSN40 (carrying a *CdARG4*, *CdHIS1* and *CmLEU2* gene, respectively [41]) were used for amplification of the selective markers and *C*. *albicans* genomic DNA was used for amplification of the 5’- and 3’-flanking sequences of the gene of interest.

To create the gene reconstituted plasmids, *CaLEU2* was amplified from *C*. *albicans* genomic DNA and inserted into plasmid pBES116 [18] at the *Pst*I/*Hind*III site, generating plasmid pBES116-LEU2. A fragment containing the sequence of a complete corresponding gene (5’-UTR + ORF +3’-UTR) was amplified and inserted into *Pst*I-digested (for *CIT1*, *ACO1*, *KGD1*, *KGD2*, *SDH3*, *SDH4*, *MDH1-1*, *FUM11*, *FUM12*, and *MLS1*), *Cla*I-digested (for *SDH2* and *MDH1-3*), or *Hind*III and *Cla*I-digested plasmid pBES116-LEU2 (*IDH1*, *IDH2*, and *ACO2*), respectively. The gene-reconstituted plasmids were then linearized with *Asc*I and transformed into the corresponding mutant to generate reconstituted strains. Linearized plasmids were integrated at the *ADE2* locus.

To construct Ras1-constitutively activated strains, plasmid pACT-RAS1V13 [61] was linearized with *Asc*I and transformed into the strain SN152, and TCA cycle mutants. Fusion PCR strategies were used to delete *PDE2* in the WT and TCA cycle mutants [41]. To delete the first allele, the mutants were transformed with the fusion PCR product of *CmLEU2* flanked by the *PDE2* 5’- and 3’-fragments. The second allele was deleted by transforming with *caSAT1*, encoding a *Candida*-optimized nourseothricin-resistant gene [62], flanked by 5’- and 3’-*PDE2* fragments. A fragment containing the ORF region of *SFL2* was sub-cloned into plasmid pACT1 [63], generating overexpressing plasmid pACT-SFL2. To construct *SFL2*-overexpressing strains, the plasmid were linearized with *Asc*I and transformed into the strain SN152. The linearized overexpressing plasmid was integrated into the *ADE2* locus. For Sfl2-ChIP assays, a 13 x Myc-tagged Sfl2-expressing strain was constructed. PCR products containing the *SFL2* ORF sequence and a C-terminal 13 x Myc-tag were prepared and subcloned into the *Eco*RV/*Kpn*I site of pACT1 [63], yielding plasmid pACT-SFL2-MYC. The strain SN152 was transformed with *Asc*I-digested pACT-SFL2-MYC, generating the 13 x Myc-tagged Sfl2 strain.

### Intracellular ATP quantification assays

Intracellular ATP concentration in *C*. *albicans* cells was determined with an ATP Bioluminescence Assay Kit (CellTiter-Glo Luminescent Cell Viability Assay, Promega, Inc.). *C*. *albicans* cells (4×10^6^) were spotted onto YPD medium and cultured at 37°C in air or in 5% CO_2_ for 16 hours. Cells were harvested and homogenized in 1x PBS with a bead beater. A standard curve was determined using serial tenfold dilutions of ATP disodium salt (Cat. #P1132, Promega, Inc.). Results of the samples were normalized to the corresponding protein concentrations, determined by the Bradford assay (BioRad, Inc.). Three independent replicates were included and the means ± standard deviations (SD) are presented. Student’s *t* tests were performed to assess the significant difference (** *p* <0.01, *p <0.05).

### Determination of intracellular cAMP levels

Intracellular cAMP levels were measured with a Monoclonal Anti-cAMP Antibody Based Direct cAMP ELISA Kit (Catalog No. 80203, NewEastBio, Inc.) following the manufacturer’s instructions. Briefly, 4×10^6^ cells of *C*. *albicans* were spotted onto YPD medium and cultured at 37°C in air or in 5% CO_2_ for 16 hours. Cells were harvested and cell pellets were immediately frozen in liquid nitrogen. For cAMP extraction, frozen cells were thawed and resuspended in 1 ml of 0.1M HCl. Half of each sample was used for the determination of cell concentration and the other half was used to measure the cAMP level with the ELISA kit. Three independent replicates were included and the means ± standard deviations (SD) are presented. Student’s *t* tests were performed to assess the significant difference (** *p* <0.01, *p <0.05).

### Determination of total Ras1 and GTP-bound Ras1 levels

Total Ras1 and GTP-bound Ras1 levels were examined according to a previous report [29]. *C*. *albicans* cells (4×10^6^) were spotted onto YPD medium and cultured at 37°C in air for 16 hours. Cells were collected and homogenized in the Lysis/Binding/Wash Buffer (Active Ras Pull-Down and Detection Kit, Pierce, Inc.). Protein concentrations were determined with the Pierce BCA Protein Assay Reagent (Product No. 23227). GTP-bound Ras1 protein was isolated using the Active Ras Pull-Down and Detection Kit (Pierce, Inc.). 500μg of total protein was used for the active Ras1 pull-down assay. 20μl of the active Ras1 isolated from the pull-down assay and 10μg of total protein (as the input control) were separated by SDS-PAGE, and then transferred to a polyvinylidene difluoride (PVDF) membrane for Western blot assays with monoclonal anti-Ras clone 10 antibody (Millipore, Inc.). α-Tubulin was used as a loading control.

### RNA extraction and quantitative reverse real-time PCR (Q-RT-PCR) assays

*C*. *albicans* cells were spotted or spread on YPD medium plates and cultured at 37°C in air or in 5% CO_2_. Cells were harvested and total RNA was extracted for Q-RT-PCR assays. Briefly, 0.6 μg of total RNA per sample was used to synthesize cDNA with RevertAid Reverse Transcriptase (Thermo Scientific, Inc.). Quantification of transcripts was performed in a Bio-Rad CFX96 real-time PCR detection system using SYBR green Mix (TOYOBO, Inc.). The expression levels of each experimental sample were normalized to that of *ACT1*.

### RNA-Seq analysis

Cells of the WT and *sfl2/sfl2* mutant were first grown at 25°C in liquid Lee’s glucose medium for 24 hours, then spread onto YPD medium plates and incubated at 37°C in air or in 5% CO_2_ for 22 hours. Colonies were harvested and total RNA was extracted as described above. RNA-Seq analysis was performed by the company Berry Genomics Co. (Beijing). Approximately 10 million (M) reads were sequenced in each library of the samples. Briefly, mRNA was purified from total RNA using Oligo(dT) magnetic beads, and fragmented into small pieces (200–700 bp). The cleaved RNA fragments were primed with random hexamers and used to synthesize the first-strand and second-strand cDNA. Sequencing adapters were ligated to the cDNA fragments. The library products were then sequenced using an Illumina HiSeq 2500 V4. Illumina software OLB_1.9.4 was used for base-calling. The raw reads were filtered by removing the adapter and low quality reads (the percentage of low quality bases with a quality value ≤3 was >50% in a read). Clean reads were mapped to the genome of *C*. *albicans* SC5314 using TopHat (version2.1.1) and Cufflinks (version2.2.1) software [64]. Mismatches less than two bases were allowed in the alignments. Relative gene expression levelswere calculated using the FPKM (Fragments Per kb per Million reads)method; FPKM = 10^6^C/(NL/10^3^), where “C” is the number of fragments that uniquely aligned to gene A, “N” represents the total number of fragments that uniquely aligned to all genes, and “L” is the number of bases of gene A. GO functional enrichment analysis was carried out according to GO terminology determined using the online CGD GO Term Finder tool (http://www.candidagenome.org/cgi-bin/GO/goTermFinder).

### Chromatin immunoprecipitation (ChIP)

The Chromatin immunoprecipitation (ChIP) protocol was adapted from Nobile *et al*. (2012) [54]. *C*. *albicans* cells were grown in liquid YPD to stationary phase at 30°C, and then collected and washed with PBS twice. 5 x 10^6^ cells were inoculated into 200 mL of YPD and cultured to OD600 = 0.4. Cells (10 mL) were transferred to a 9 mm-dish and treated in 5% CO_2_ at 37°C with shaking for 6 hours, and then fixed and cross-linked with 1% formaldehyde at room temperature. The cross-linking reaction was quenched after 20 min by adding glycine to a final concentration of 125 mM. The cells were harvested, resuspended in ice-cold lysis buffer, and homogenized with a bead beater. Sonication was performed with a Diagenode Biorupter (15 min, high setting, 30 sec on, 1 min off) to obtain chromatin fragments of an average size of 500–1000 bp. The chromatin was immunoprecipitated with 2 μg of anti-Myc antibody (Millipore, Inc.) and protein A-Sepharose beads (GE Healthcare). ChIP DNA was analyzed by quantitative real-time PCR assays.

### Accession number

The RNA-seq dataset has been deposited into the NCBI Gene Expression Omnibus (GEO) portal (accession# GSE102039).

## Supporting information

S1 Fig Roles of the TCA cycle and glyoxylate bypass in utilization of different carbon sources in *C*. *albicans*. Red rectangles highlight strains that exhibited no obvious growth; purple rectangles highlight the strains that exhibited severe growth defects. *CaLEU2* was reintroduced to all the mutants. The control strain (WT) is SN152+. Cells of the WT and 15 mutants were diluted to 1 x 10^8^ cells/mL. Ten-fold serial dilutions (5 μL) were spotted onto different media and cultured at 37°C for four days. YNB, yeast nitrogen base containing 5 g/L of (NH4)_2_SO_4_. YNB+7AAs, medium containing YNB and seven amino acids (A, R, Q, E, N, P, and S). YNB+Glucose (2%), YNB+GlcNAc (2%),YNB+Sucrose (2%), YNB+Fructose (2%), YNB+glycerol (2%), YNB+ethanol (2%).
(TIF)

S2 Fig Growth curves of the WT, *cit1/cit1*, *aco1/aco1*, *idh1/idh1*, *idh2/idh2*, *kgd1/kgd1*, *kgd2/kgd2*, *sdh2/sdh2*, *sdh3/sdh3*, and *mdh1-1/mdh1-1* mutants of *C*. *albicans* in liquid media. Cells were first grown in liquid YPD to stationary phase at 30°C and then collected and washed with 1 x PBS twice. 6 x 10^6^ cells were inoculated into 3 mL of each medium as indicated. Cell densities were detected at different time points. Three independent repeats were performed. Error bars represent standard deviation (SD). *CaLEU2* was reintroduced to all the mutants. (A) Growth curves of the WT(SN152+) and mutants in liquid YPD, YNB+2% glucose, YNB+2% glycerol and YNB+2% ethanol media at 37°C. (B) Growth curves of the WT and mutants in liquid YPD, YNB+2% glucose, and YNB+2% glycerol media at 30°C.
(TIF)

S3 Fig Growth curves of the WT, *mls1/mls1*, *sdh4/sdh4*, *aco2/aco2*, *fum11/fum11*, *fum12/fum12*, and *mdh1-3/mdh1-3* mutants of *C*. *albicans* in liquid media. This figure is related to S2 Fig and the same culture conditions and data analyses were used. *CaLEU2* was reintroduced to all the mutants. (A) Growth curves of the WT(SN152+) and mutants in liquid YPD, YNB+2% glucose, YNB+2% glycerol and YNB+2% ethanol media at 37°C. (B) Growth curves of the WT and mutants in liquid YPD, YNB+2% glucose, and YNB+2% glycerol media at 30°C.
(TIF)

S4 Fig Hyphal growth of the TCA and glyoxylate cycle mutants of *C*. *albicans* in solid and liquid media containing fetal bovine serum. Cells of different mutants were plated onto YPD + serum solid medium and incubated at 37°C in air for three days. For liquid cultures, cells were inoculated in YPD + 10% serum medium and incubated at 37°C in air for three hours. Scale bars: 1mm for colony images; 20 μm for cellular images. The number of “+” signs, indicates the degree of hyphal growth. “-”indicates that no hyphal growth was observed. The percentage of hyphal cells observed is shown in each image. The control strain (WT) is SN152+.
(TIF)

S5 Fig Cellular morphologies of the WT, TCA and glyoxylate cycle mutants grown on YPD medium in air or in 5% CO_2_. Green rectangles highlight the strains that exhibited hyphal growth defects. The control strain (WT) is SN152. This figure is related to Fig 2.
(TIF)

S6 Fig Hyphal growth of the reconstituted strains of TCA and glyoxylate cycle mutants in air or in 5% CO_2_. Cells of the reconstituted strains were plated onto YPD medium and grown at 37°C in air or in 5% CO_2_ for three days. Scale bar: 1 mm. Related to Fig 2.
(TIF)

S7 Fig Effect of activating the Ras1-cAMP/PKA signaling pathway in TCA cycle mutants on hyphal growth in air. (A) Overexpression of *RAS1V13*, encoding the activating form of Ras1, in SN152, *kgd1/kgd1*, *kgd2/kgd2*, and *sdh2/sdh2* mutants. (B) Disruption of the high affinity cyclic nucleotide phosphodiesterase encoding gene, *PDE2*, in SN152, *idh1/idh1*, *idh2/idh2*, *sdh2/sdh2*, *sdh3/sdh3*, and *mdh1-1/mdh1-1* strains. Colony and cellular images are shown. Scale bar: 1 mm for colony images; 20 μm for cellular images.
(TIF)

S8 Fig Effect of activating the Ras1-cAMP/PKA signaling pathway in TCA cycle mutants on hyphal growth in 5% CO_2_. (A) Overexpression of *RAS1V13*, encoding the activating form of Ras1, in SN152, *kgd1/kgd1*, *kgd2/kgd2*, and *sdh2/sdh2* mutants. (B) Disruption of the high affinity cyclic nucleotide phosphodiesterase encoding gene *PDE2* in SN152, *idh1/idh1*, *idh2/idh2*, *sdh2/sdh2*, *sdh3/sdh3*, and *mdh1-1/mdh1-1* mutants. Colony and cellular images are shown. The control strain (WT) is SN152. Scale bars: 1 mm for colony images; 20 μm for cellular images. Related to S7 Fig.
(TIF)

S1 Table Growth rate of the TCA gene mutants in *C*. *albicans* at 37°C. (DOCX)

S2 Table Strains used in this study. (DOC)

S3 Table Primers used in this study. (DOCX)

S1 Dataset RNA-Seq dataset. (XLSX)
