# Supplementary figures and images for: Integration of the tricarboxylic acid (TCA) cycle with cAMP signaling and Sfl2 pathways in the regulation of CO2 sensing and hyphal development in Candida albicans
Source: PLoS Genet. 2017 Aug 7;13(8):e1006949. doi: 10.1371/journal.pgen.1006949 (PMC5567665; doi:10.1371/journal.pgen.1006949)

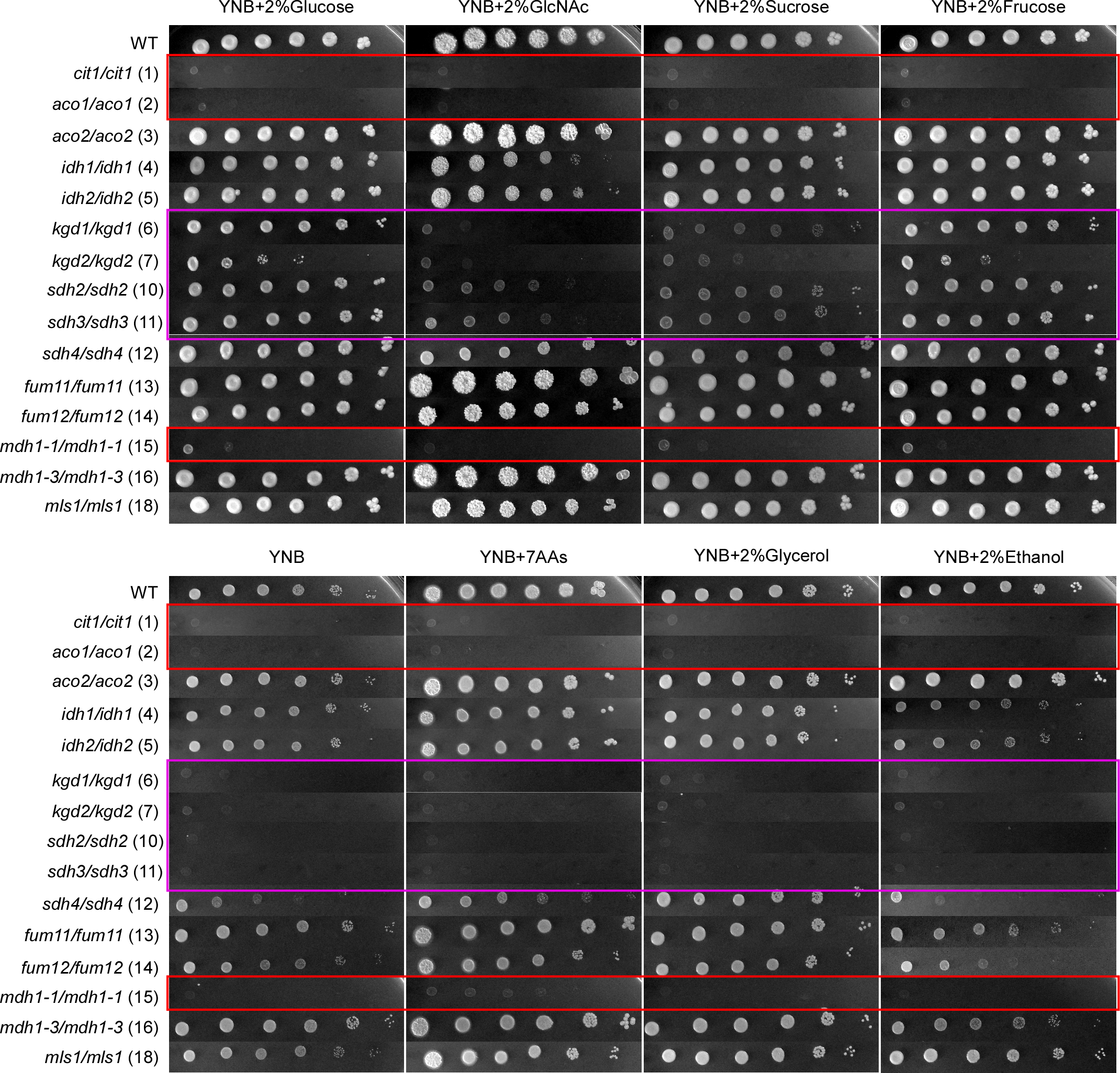

Supplement: S1 Fig — Red rectangles highlight strains that exhibited no obvious growth; purple rectangles highlight the strains that exhibited severe growth defects. CaLEU2 was reintroduced to all the mutants. The control strain (WT) is SN152+. Cells of the WT and 15 mutants were diluted to 1 x 10^8^ cells/mL. Ten-fold serial dilutions (5 μL) were spotted onto different media and cultured at 37°C for four days. YNB, yeast nitrogen base containing 5 g/L of (NH4)2SO4. YNB+7AAs, medium containing YNB and seven amino acids (A, R, Q, E, N, P, and S). YNB+Glucose (2%), YNB+GlcNAc (2%),YNB+Sucrose (2%), YNB+Fructose (2%), YNB+glycerol (2%), YNB+ethanol (2%). (TIF) [file pgen.1006949.s001.tif]

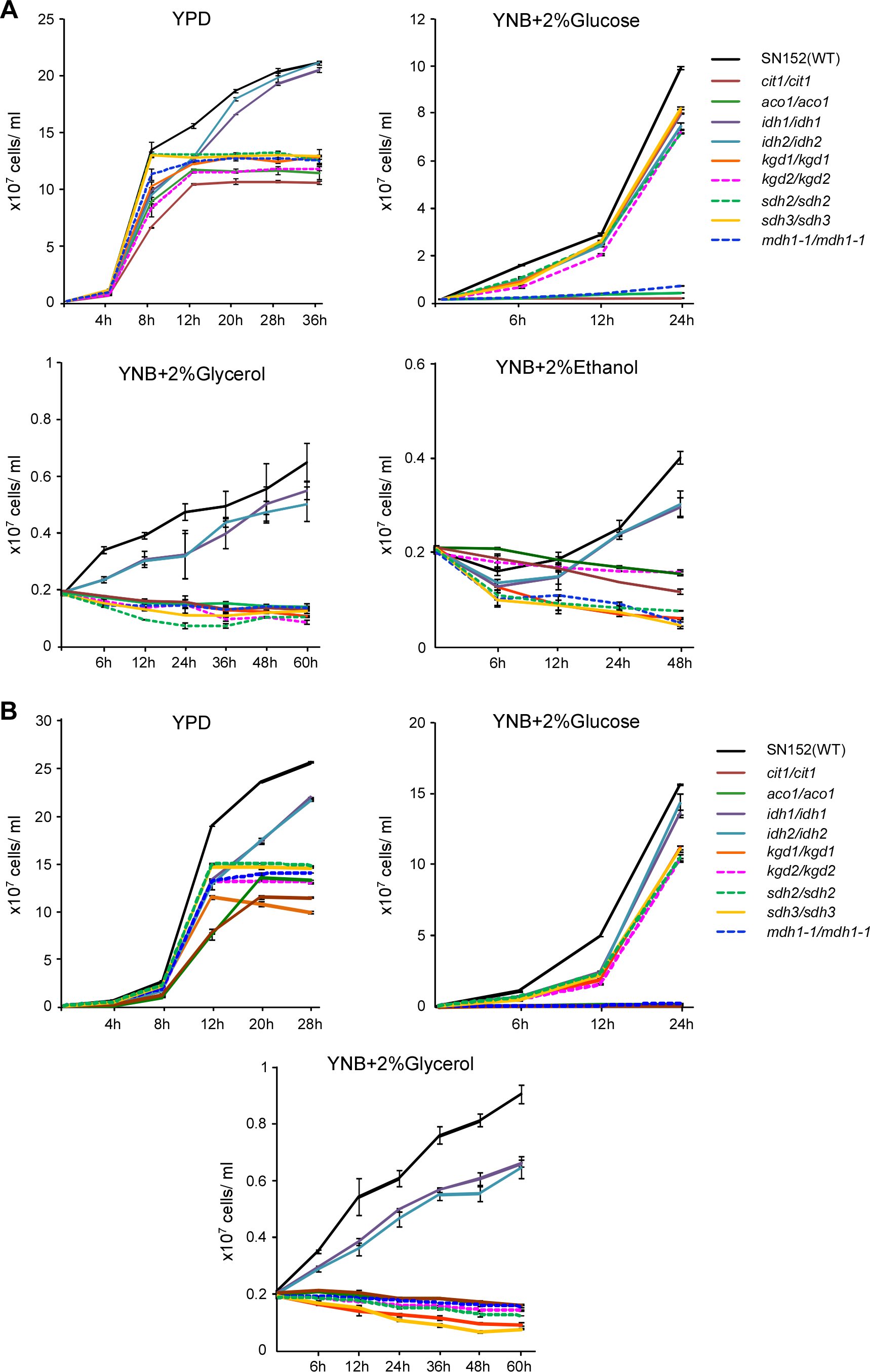

Supplement: S2 Fig — Cells were first grown in liquid YPD to stationary phase at 30°C and then collected and washed with 1 x PBS twice. 6 x 10^6^ cells were inoculated into 3 mL of each medium as indicated. Cell densities were detected at different time points. Three independent repeats were performed. Error bars represent standard deviation (SD). CaLEU2 was reintroduced to all the mutants. (A) Growth curves of the WT(SN152+) and mutants in liquid YPD, YNB+2% glucose, YNB+2% glycerol and YNB+2% ethanol media at 37°C. (B) Growth curves of the WT and mutants in liquid YPD, YNB+2% glucose, and YNB+2% glycerol media at 30°C. (TIF) [file pgen.1006949.s002.tif]

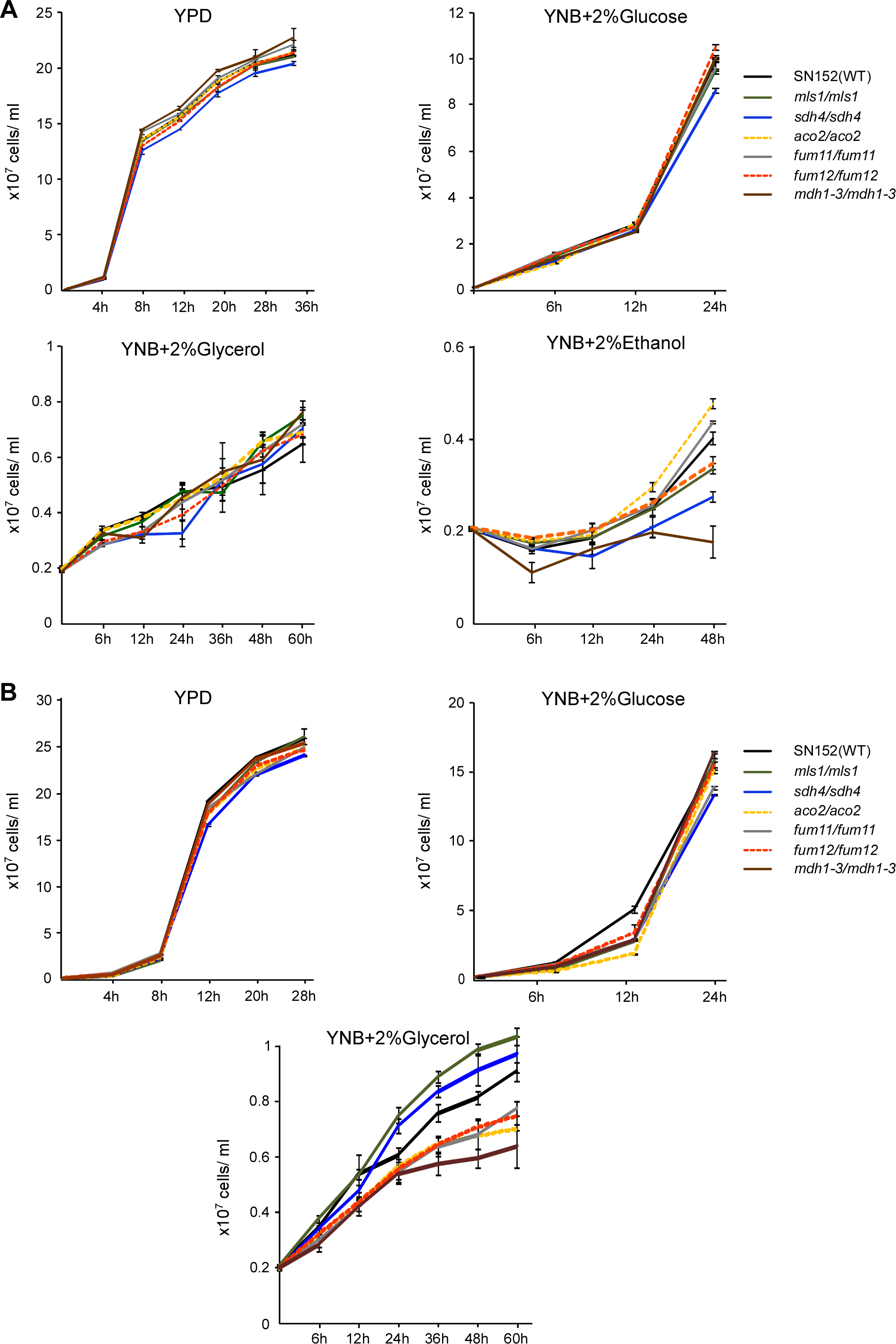

Supplement: S3 Fig — This figure is related to S2 Fig and the same culture conditions and data analyses were used. CaLEU2 was reintroduced to all the mutants. (A) Growth curves of the WT(SN152+) and mutants in liquid YPD, YNB+2% glucose, YNB+2% glycerol and YNB+2% ethanol media at 37°C. (B) Growth curves of the WT and mutants in liquid YPD, YNB+2% glucose, and YNB+2% glycerol media at 30°C. (TIF) [file pgen.1006949.s003.tif]

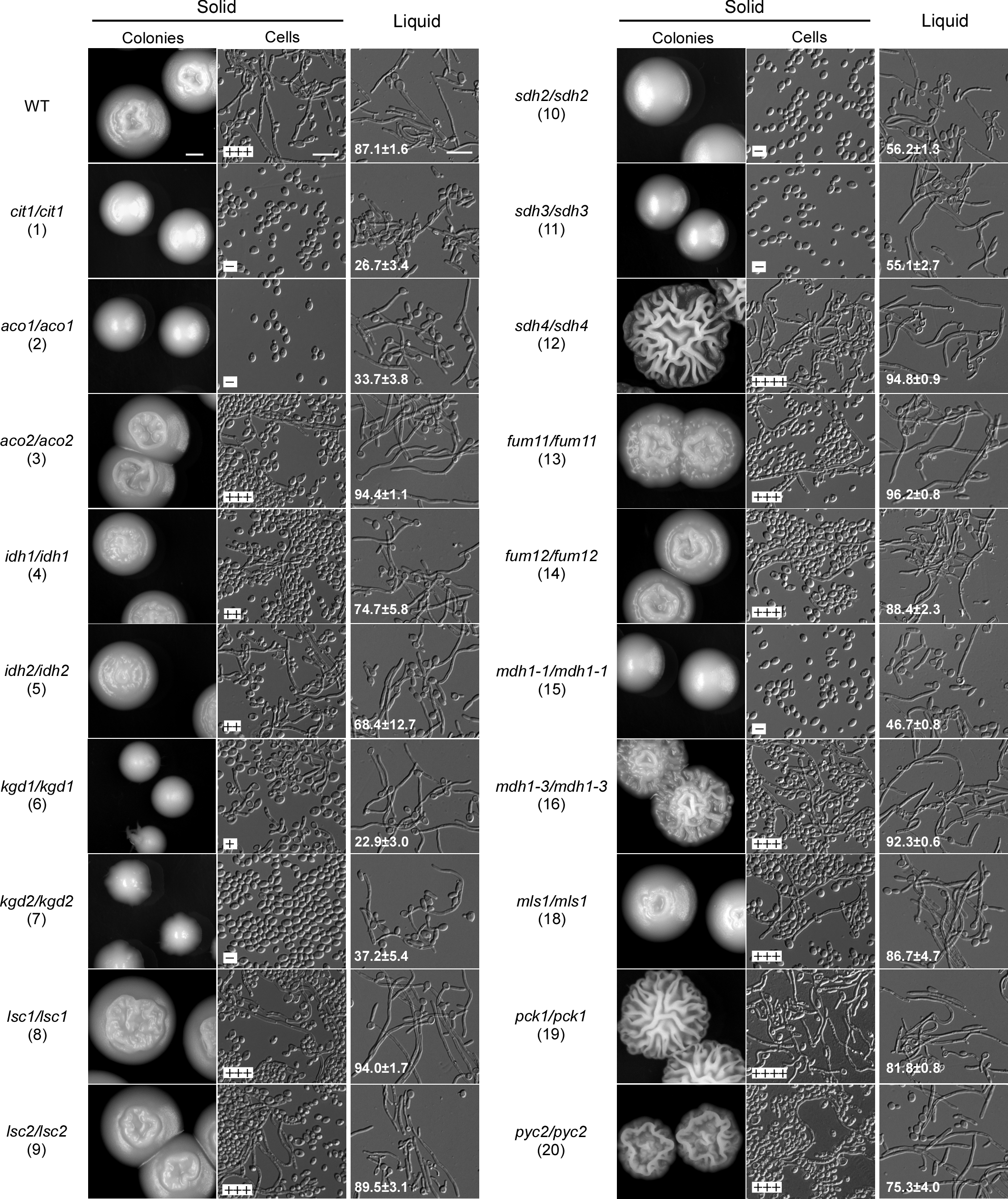

Supplement: S4 Fig — Cells of different mutants were plated onto YPD + serum solid medium and incubated at 37°C in air for three days. For liquid cultures, cells were inoculated in YPD + 10% serum medium and incubated at 37°C in air for three hours. Scale bars: 1mm for colony images; 20 μm for cellular images. The number of “+” signs, indicates the degree of hyphal growth. “-”indicates that no hyphal growth was observed. The percentage of hyphal cells observed is shown in each image. The control strain (WT) is SN152+. (TIF) [file pgen.1006949.s004.tif]

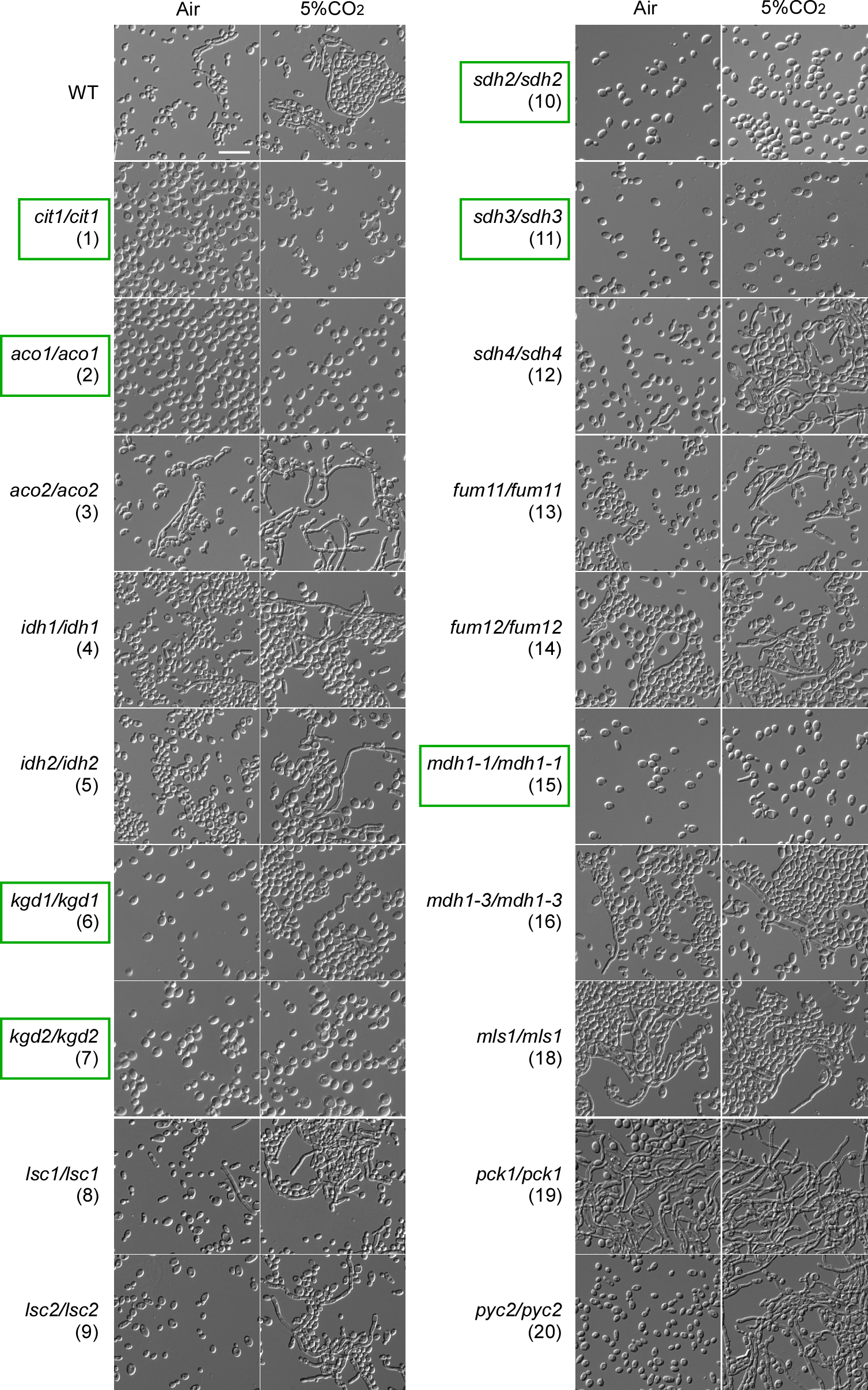

Supplement: S5 Fig — Green rectangles highlight the strains that exhibited hyphal growth defects. The control strain (WT) is SN152. This figure is related to Fig 2. (TIF) [file pgen.1006949.s005.tif]

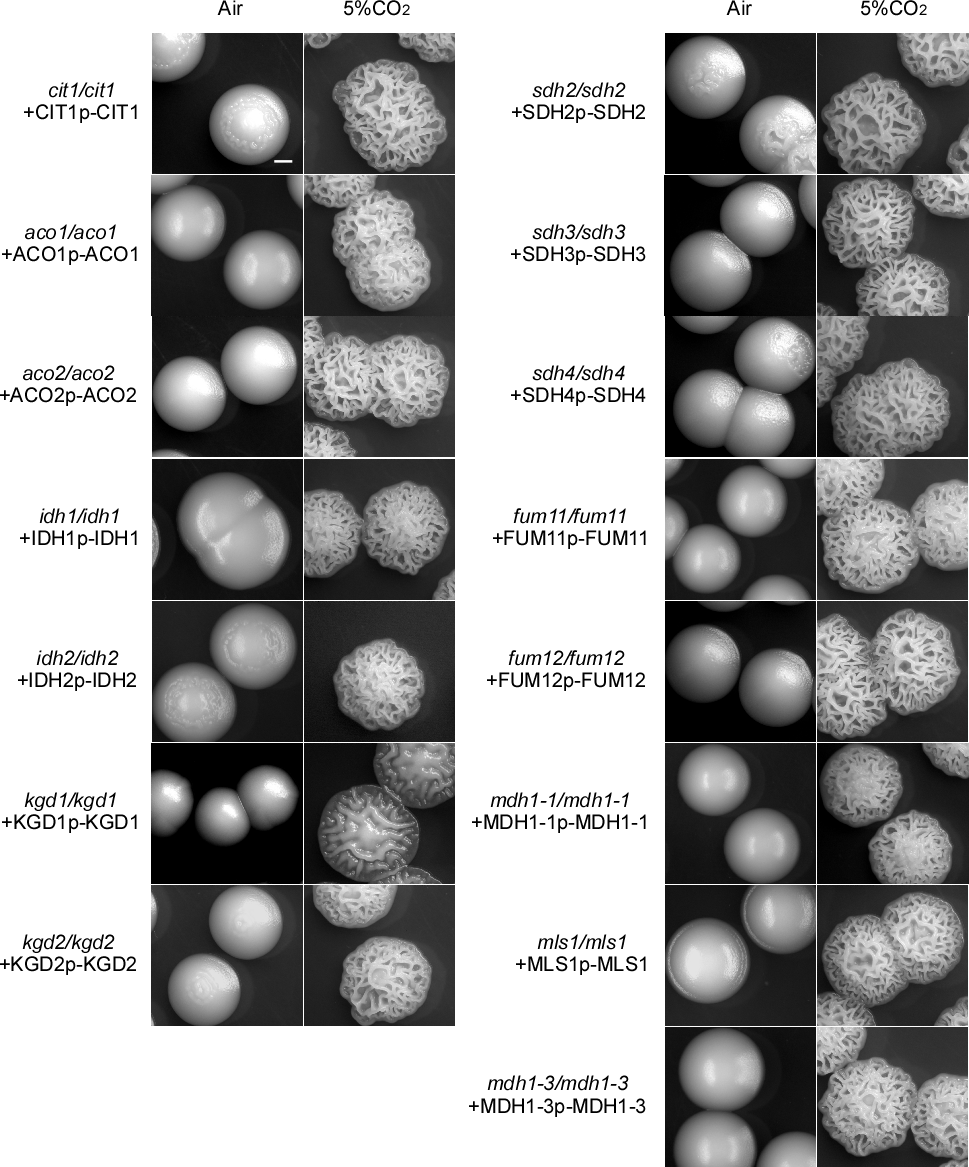

Supplement: S6 Fig — Cells of the reconstituted strains were plated onto YPD medium and grown at 37°C in air or in 5% CO2 for three days. Scale bar: 1 mm. Related to Fig 2. (TIF) [file pgen.1006949.s006.tif]

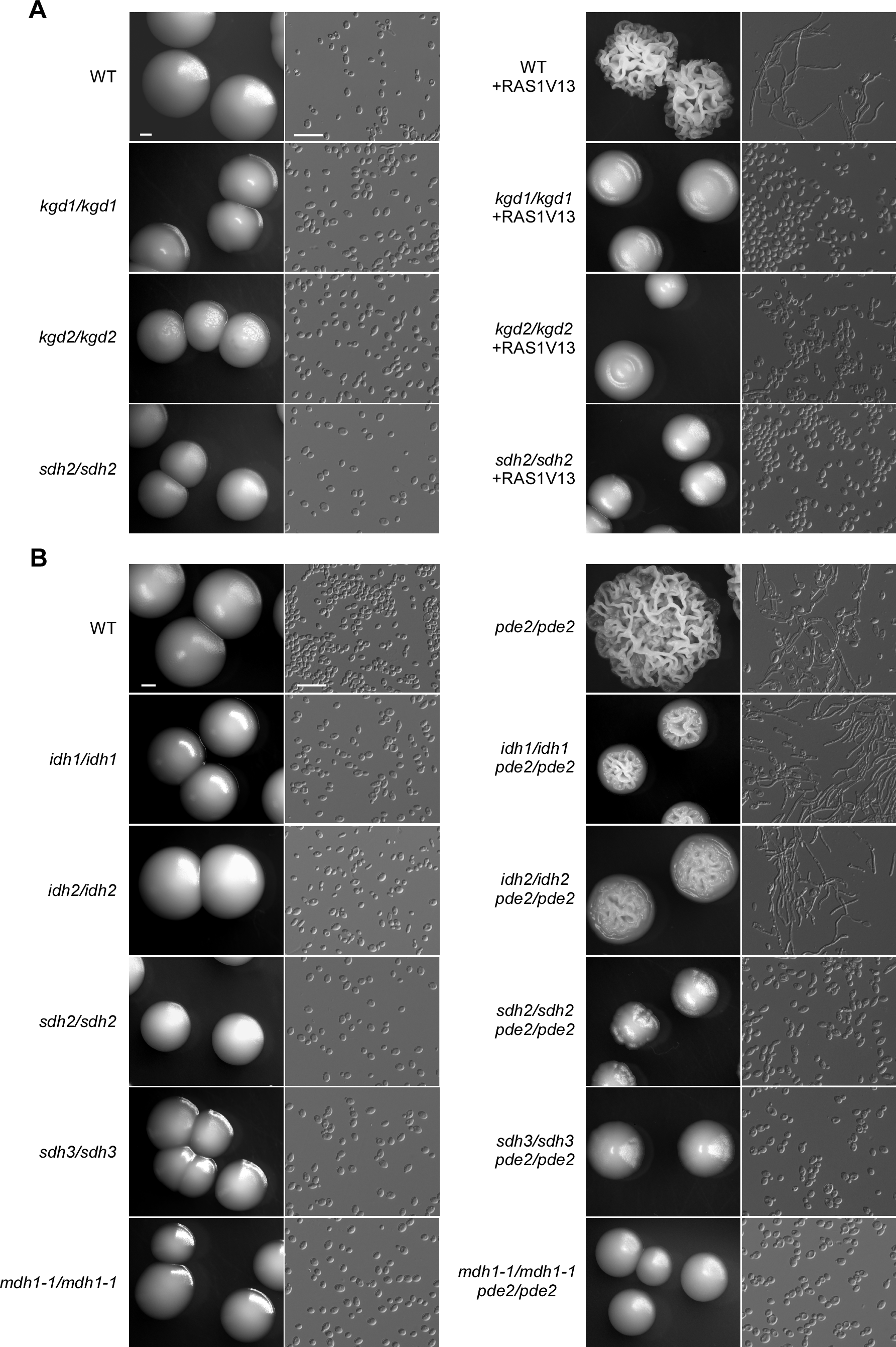

Supplement: S7 Fig — (A) Overexpression of RAS1V13, encoding the activating form of Ras1, in SN152, kgd1/kgd1, kgd2/kgd2, and sdh2/sdh2 mutants. (B) Disruption of the high affinity cyclic nucleotide phosphodiesterase encoding gene, PDE2, in SN152, idh1/idh1, idh2/idh2, sdh2/sdh2, sdh3/sdh3, and mdh1-1/mdh1-1 strains. Colony and cellular images are shown. Scale bar: 1 mm for colony images; 20 μm for cellular images. (TIF) [file pgen.1006949.s007.tif]

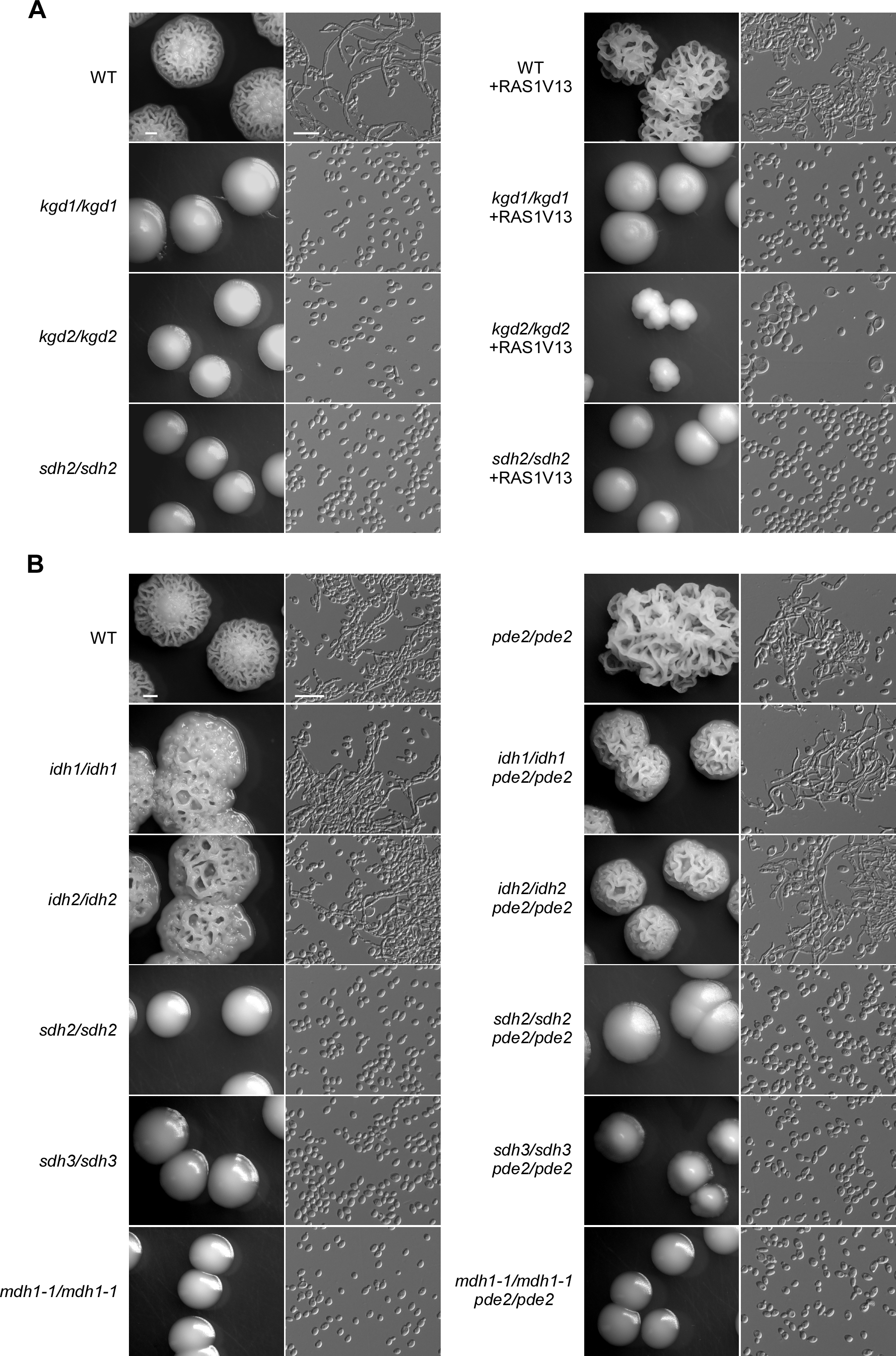

Supplement: S8 Fig — (A) Overexpression of RAS1V13, encoding the activating form of Ras1, in SN152, kgd1/kgd1, kgd2/kgd2, and sdh2/sdh2 mutants. (B) Disruption of the high affinity cyclic nucleotide phosphodiesterase encoding gene PDE2 in SN152, idh1/idh1, idh2/idh2, sdh2/sdh2, sdh3/sdh3, and mdh1-1/mdh1-1 mutants. Colony and cellular images are shown. The control strain (WT) is SN152. Scale bars: 1 mm for colony images; 20 μm for cellular images. Related to S7 Fig. (TIF) [file pgen.1006949.s008.tif]
