# Supplementary material for: Integration of the tricarboxylic acid (TCA) cycle with cAMP signaling and Sfl2 pathways in the regulation of CO2 sensing and hyphal development in Candida albicans
Source: PLoS Genet. 2017 Aug 7;13(8):e1006949. doi: 10.1371/journal.pgen.1006949 (PMC5567665; doi:10.1371/journal.pgen.1006949)
Supplement: S1 Table — (DOCX) [file pgen.1006949.s009.docx]

**Table S1. Growth rate of the TCA gene mutants in *C. albicans* at 37^o^C.**

| Strains | Growth rate | | | | Hyphal  development | |
| --- | --- | --- | --- | --- | --- | --- |
|  | YPD | YNB +  Glucose | YNB +  Glycerol | YNB+  Ethanol |  | YPD + Serum |
| WT | +++++ | ++++ | +++ | ++ |  | +++ |
| *cit1/cit1* (1) | ++ | G.d. | G.d. | G.d. |  | - |
| *aco1/aco1* (2) | +++ | G.d. | G.d. | G.d. |  | - |
| *aco2/aco2* (3) | +++++ | ++++ | +++ | ++ |  | +++ |
| *idh1/idh1* (4) | ++++ | +++ | ++ | + |  | ++ |
| *Idh2/idh2* (5) | ++++ | +++ | ++ | + |  | ++ |
| *kgd1/kgd1* (6) | +++ | +++ | G.d. | G.d. |  | + |
| *Kgd2/kgd2* (7) | +++ | ++ | G.d. | G.d. |  | - |
| *sdh2/sdh2* (10) | +++ | +++ | G.d. | G.d. |  | - |
| *sdh3/sdh3* (11) | +++ | +++ | G.d. | G.d. |  | - |
| *sdh4/sdh4* (12) | +++++ | +++ | ++ | + |  | ++++ |
| *fum11/fum11* (13) | +++++ | ++++ | +++ | ++ |  | +++ |
| *fum12/fum12* (14) | +++++ | ++++ | +++ | ++ |  | +++ |
| *mdh1-1/mdh1-1* (15) | +++ | G.d. | G.d. | G.d. |  | - |
| *mdh1-3/mdh1-3* (16) | +++++ | ++++ | +++ | + |  | +++ |
| *mls1/mls1* (18) | +++++ | ++++ | +++ | ++ |  | +++ |

G. d.: Growth defect. The number of “+” represented the growth rate of different mutants. “-” represented no filamentous growth was observed.

For cell growth rate assays, cells were first grown in liquid YPD to stationary phase at 30°C and then collected and washed with 1 x PBS twice. 2 x 10^6^ cells were inoculated into 3 mL of each medium as indicated. Cell densities were detected at different time points. Three independent repeats were performed. Media used for growth rate examination: YPD, YNB+2% glucose, YNB+2% glycerol and YNB+2% ethanol media at 37°C.

Hyphal assays were performed in YPD + 10% serum medium at 37°C.
