# Supplementary material for: Integration of the tricarboxylic acid (TCA) cycle with cAMP signaling and Sfl2 pathways in the regulation of CO2 sensing and hyphal development in Candida albicans
Source: PLoS Genet. 2017 Aug 7;13(8):e1006949. doi: 10.1371/journal.pgen.1006949 (PMC5567665; doi:10.1371/journal.pgen.1006949)
Supplement: S2 Table — (DOC) [file pgen.1006949.s010.doc]

**Table S2. Strains used in this study**

| **Strain name** | **Parent strain** | **Genotype** | **Purpose** | **Ref.** |
| --- | --- | --- | --- | --- |
| SN152 | SC5314 | MTL**a**/α ura3:: λimm434::URA3-IRO1/ura3:: λimm434, arg4::hisG/arg4::hisG his1::hisG/his1::hisG leu2::hisG/leu2::hisG | Morphological analysis | [1] |
| SN152+ | SN152 | As SN152, but *his*1::hisG/HIS1 *arg4*::hisG/ARG4 *leu2*::hisG/LEU2 | Carbon source utilization & Virulence | This study |
| *cit1/cit1* | SN152 | As SN152, but *cit1::ARG4/cit1::HIS1* | Morphological analysis | This study |
| *aco1/aco1* |  | As SN152, but *aco1::ARG4/aco1::HIS1* |  |  |
| *aco2/aco2* |  | As SN152, but *aco2::ARG4/aco2::HIS1* |  |  |
| *idh1/idh1* |  | As SN152, but *idh1::ARG4/idh1::HIS1* |  |  |
| *idh2/idh2* |  | As SN152, but *idh2::ARG4/idh2::HIS1* |  |  |
| *kgd1/kgd1* |  | As SN152, but *kgd1::ARG4/kgd1::HIS1* |  |  |
| *kgd2/kgd2* |  | As SN152, but *kgd2::ARG4/kgd2::HIS1* |  |  |
| *sdh2/sdh2* |  | As SN152, but *sdh2::ARG4/sdh2::HIS1* |  |  |
| *sdh3/sdh3* |  | As SN152, but *sdh3::ARG4/sdh3::HIS1* |  |  |
| *sdh4/sdh4* |  | As SN152, but *sdh4::ARG4/sdh4::HIS1* |  |  |
| *fum11/fum11* |  | As SN152, but *fum11::ARG4/fum11::HIS1* |  |  |
| *fum12/fum12* |  | As SN152, but *fum12::ARG4/fum12::HIS1* |  |  |
| *mdh1-1/mdh1-1* |  | As SN152, but *mdh1-1::ARG4/mdh1-1::HIS1* |  |  |
| *mls1/mls1* |  | As SN152, but *mls1::ARG4/mls1::HIS1* |  |  |
| *mdh1-3/mdh1-3* |  | As SN152, but *mdh1-3::ARG4/mdh1-3::HIS1* |  |  |
| *lsc1/lsc1* |  | As SN152, but *isc1::ARG4/isc1::HIS1* |  |  |
| *lsc2/lsc2* |  | As SN152, but *isc2::ARG4/isc2::HIS1* |  |  |
| *pck1/pck1* |  | As SN152, but *pck1::ARG4/pck1::LEU2* |  |  |
| *pyc2/pyc2* |  | As SN152, but *pyc2::ARG4/pyc2::LEU2* |  |  |
| *cit1/cit1*+ | *cit1/cit1* | As *cit1/cit1*, but *leu2::hisG/LEU2* | Carbon source utilization & Virulence | This study |
| *aco1/aco1*+ | *aco1/aco1* | As *aco1/aco1*, but *leu2::hisG/LEU2* |  |  |
| *aco2/aco2*+ | *aco2/aco2* | As *aco2/aco2*, but *leu2::hisG/LEU2* |  |  |
| *idh1/idh1*+ | *idh1/idh1* | As *idh1/idh1*, but *leu2::hisG/LEU2* |  |  |
| *idh2/idh2*+ | *idh2/idh2* | As *idh2/idh2*, but *leu2::hisG/LEU2* |  |  |
| *kgd1/kgd1*+ | *kgd1/kgd1* | As *kgd1/kgd1*, but *leu2::hisG/LEU2* |  |  |
| *kgd2/kgd2*+ | *kgd2/kgd2* | As *kgd2/kgd2*, but *leu2::hisG/LEU2* |  |  |
| *sdh2/sdh2*+ | *sdh2/sdh2* | As *sdh2/sdh2*, but *leu2::hisG/LEU2* |  |  |
| *sdh3/sdh3*+ | *sdh3/sdh3* | As *sdh3/sdh3*, but *leu2::hisG/LEU2* |  |  |
| *sdh4/sdh4*+ | *sdh4/sdh4* | As *sdh4/sdh4*, but *leu2::hisG/LEU2* |  |  |
| *fum11/fum11*+ | *fum11/fum11* | As *fum11/fum11*, but *leu2::hisG/LEU2* |  |  |
| *fum12/fum12*+ | *fum12/fum12* | As *fum12/fum12*, but *leu2::hisG/LEU2* |  |  |
| *mdh1-1/mdh1-1*+ | *mdh1-1/mdh1-1* | As *mdh1-1/mdh1-1*, but *leu2::hisG/LEU2* |  |  |
| *mls1/mls1*+ | *mls1/mls1* | As *mls1/mls1*, but *leu2::hisG/LEU2* |  |  |
| *mdh1-3/mdh1-3*+ | *mdh1-3/mdh1-3* | As *mdh1-3/mdh1-3*, but *leu2::hisG/LEU2* |  |  |
| *cit1/*CIT1p-CIT1 | *cit1/cit1* | As *cit1/cit1*, but CIT1p-CIT1-LEU2 | Morphological analysis and virulence | This study |
| *aco1/* ACO1p-ACO1 | *aco1/aco1* | As *aco1/aco1*, but ACO1p-ACO1-LEU2 |  |  |
| *aco2/* ACO2p-ACO2 | *aco2/aco2* | As *aco2/aco2*, but ACO2p-ACO2-LEU2 |  |  |
| *idh1/* IDH1p-IDH1 | *idh1/idh1* | As *idh1/idh1*, but IDH1p-IDH1-LEU2 |  |  |
| *idh2/* IDH2p-IDH2 | *idh2/idh2* | As *idh2/idh2*, but IDH2p-IDH2-LEU2 |  |  |
| *kgd1/* KGD1p-KGD1 | *kgd1/kgd1* | As *kgd1/kgd1*, but KGD1p-KGD1-LEU2 |  |  |
| *kgd2/* KGD2p-KGD2 | *kgd2/kgd2* | As *kgd2/kgd2*, but KGD2p-KGD2-LEU2 |  |  |
| *sdh2/* SDH2p-SDH2 | *sdh2/sdh2* | As *sdh2/sdh2*, but SDH2p-SDH2-LEU2 |  |  |
| *sdh3/* SDH3p-SDH3 | *sdh3/sdh3* | As *sdh3/sdh3*, but SDH3p-SDH3-LEU2 |  |  |
| *sdh4/* SDH4p-SDH4 | *sdh4/sdh4* | As *sdh4/sdh4*, but SDH4p-SDH4-LEU2 |  |  |
| *fum11/* FUM11p-FUM11 | *fum11/fum11* | As *fum11/fum11*, but FUM11p-FUM11-LEU2 |  |  |
| *fum12/* FUM12p-FUM12 | *fum12/fum12* | As *fum12/fum12*, but FUM12p-FUM12-LEU2 |  |  |
| *mdh1-1/* MDH1-1p-MDH1-1 | *mdh1-1/mdh1-1* | As *mdh1-1/mdh1-1*, but MDH1-1p-MDH1-1-LEU2 |  |  |
| *mls1/* MLS1p-MLS1 | *mls1/mls1* | As *mls1/mls1*, but MLS1p-MLS1-LEU2 |  |  |
| *mdh1-3/* MDH1-3p-MDH1-3 | *mdh1-3/mdh1-3* | As *mdh1-3/mdh1-3*, but MDH1-3p-MDH1-3-LEU2 |  |  |
| *ras1/ras1* | CAI4 | MTL**a**/α *ura3::imm434/ura3::imm434, iro1/iro1::imm434, ras1::FTR/ras1::FTR* | Morphological analysis & gene regulation | [2] |
| *cyr1/cyr1* | CAI4 | MTL**a**/α *cyr1::FTR/cyr1::FTR* |  |  |
| *tpk1/tpk1* | SN152 | As SN152, *tpk1::HIS1/ tpk1::ARG4* |  | [4] |
| *tpk2/tpk2* | SN152 | As SN152, *tpk2::HIS1/ tpk2::ARG4* |  |  |
| *tpk2/tpk2 tpk1/tpk1 (t2t1)* | *tpk2/tpk2* | As tpk2/tpk2, but *tpk1::LEU2/ tpk1::FRT* |  |  |
| WT+RAS1V13 | SN152 | As SN152, but p ACT-RAS1V13-LEU2 | Morphological analysis | This study |
| *kgd1/kgd1*+RAS1V13 | *kgd1/kgd1* | As *kgd1/kgd1*, but pACT-RAS1V13-LEU2 |  |  |
| *kgd2/kgd2*+RAS1V13 | *kgd2/kgd2* | As *kgd2/kgd2*, but pACT-RAS1V13-LEU2 |  |  |
| *sdh2/sdh2*+RAS1V13 | *sdh2/sdh2* | As *sdh2/sdh2*, but pACT-RAS1V13-LEU2 |  |  |
| *idh1/idh1 pde2/pde2* | *idh1/idh1* | As *idh1/idh1*, but *pde2::LEU2/ pde2::SAT1* |  |  |
| *idh2/idh2 pde2/pde2* | *idh2/idh2* | As *idh2/idh2*, but *pde2::LEU2/ pde2::SAT1* |  |  |
| *sdh2/sdh2 pde2/pde2* | *sdh2/sdh2* | As *sdh2/sdh2*, but *pde2::LEU2/ pde2::SAT1* |  |  |
| *sdh3/sdh3 pde2/pde2* | *sdh3/sdh3* | As *sdh3/sdh3*, but *pde2::LEU2/ pde2::SAT1* |  |  |
| *mdh1-1/mdh1-1 pde2/pde2* | *mdh1-1/mdh1-1* | As *mdh1-1/mdh1-1*, but *pde2::LEU2/ pde2::SAT1* |  |  |
| *efg1/efg1* | SN152 | As SN152, but *efg1::HIS1/ efg1::LEU2* | Morphological analysis & gene regulation | [3] |
| *flo8/flo8* |  | As SN152, but *flo8::HIS1/ flo8::LEU2* |  |  |
| *brg1/brg1* |  | As SN152, but *brg1::HIS1/ brg1::LEU2* |  |  |
| *ndt80/ndt80* |  | As SN152, but *ndt80::HIS1/ ndt80::LEU2* |  |  |
| *sfl2/sfl2* |  | As SN152, but *sfl2::HIS1/ sfl2::LEU2* |  |  |
| WT+pACT-SFL2 |  | As SN152, but A*DE2/ade2::*pACT-SFL2-URA3 |  | This study |
| WT+pACT-EFG1 |  | As SN152, but A*DE2/ade2::* pACT-EFG1-URA3 |  | [4] |
| WT+pACT-BRG1 |  | As SN152, but A*DE2/ade2::*pACT-BRG1-URA3 |  |  |
| WT+pACT-SFL2 |  | As SN152, but A*DE2/ade2::*pACT-SFL2-URA3 |  |  |
| WT+pACT-TPK1 |  | As SN152, but A*DE2/ade2::*pACT-TPK1-URA3 |  |  |
| WT+pACT-TPK2 |  | As SN152, but A*DE2/ade2::*pACT-TPK2-URA3 |  |  |
| WT+pACT-SFL2-MYC |  | As SN152, but A*DE2/ade2::*pACT-SFL2-MYC-URA3 | ChIP assay | This study |

**References**

1. Noble SM, Johnson AD (2005) Strains and strategies for large-scale gene deletion studies of the diploid human fungal pathogen *Candida albicans*. Eukaryot Cell 4: 298-309.

2. Yi S, Sahni N, Daniels KJ, Lu KL, Srikantha T, Huang G, Garnaas AM, Soll DR (2011) Alternative mating type configurations (a/α versus a/a or α/α) of *Candida albicans* result in alternative biofilms regulated by different pathways. PLoS Biol 9(8):e1001117.

3. Homann OR, Dea J, Noble SM, Johnson AD (2009) A phenotypic profile of the *Candida albicans* regulatory network. PLoS Genet 5: e1000783.

# 4. Cao C, Wu M, Bing J, Tao L, Ding X, Liu X, Huang G (2017) Global regulatory roles of the cAMP/PKA pathway revealed by phenotypic, transcriptomic and phosphoproteomic analyses in a null mutant of the PKA catalytic subunit in *Candida albicans.* Mol Microbiol doi:10.1111/mmi.13681.
