# Supplementary material for: Integration of the tricarboxylic acid (TCA) cycle with cAMP signaling and Sfl2 pathways in the regulation of CO2 sensing and hyphal development in Candida albicans
Source: PLoS Genet. 2017 Aug 7;13(8):e1006949. doi: 10.1371/journal.pgen.1006949 (PMC5567665; doi:10.1371/journal.pgen.1006949)
Supplement: S3 Table — (DOCX) [file pgen.1006949.s011.docx]

**Table S3. Primers used in this study**

| **Name** | **Sequence (5’ to 3’)** | **Purpose** |
| --- | --- | --- |
| Marker-F | CCGCTGCTAGGCGCGCCGTGACCAGTGTGATGGATATCTGC | Amplification for *ARG4, HIS1, LEU2, URA3* fragments used in fusion PCR |
| Marker-R | GCAGGGATGCGGCCGCTGACAGCTCGGATCCACTAGTAACG |  |
| ARG4-CHF | TGCATTGACTACAGTGGAAC | Gene KO confirmation  (first copy) |
| ARG4-CHR | ATCATGCCATTCTTGTCTG |  |
| HIS1-CHF | ATTAGATACGTTGGTGGTTC |  |
| HIS1-CHR | AACACAACTGCACAATCTGG |  |
| LEU2-CHF | AGAATTCCCAACTTTGTCTG |  |
| LEU2-CHR | AAACTTTGAACCCGGCTGCG |  |
| CIT1-L Fwd | AACGTGATGCTGGTTCTGTC | Fusion PCR for *CIT1* KO |
| CIT1-L rev | CACGGCGCGCCTAGCAGCGGTGCTGTCCGTACAACTTTACC |  |
| CIT1-R Fwd | GTCAGCGGCCGCATCCCTGCTTGGAAACTATTGGTGGTGC |  |
| CIT1-R rev | TTGATTATCGGGTGGTTAGG |  |
| CIT1-CHF | TTGTTTGATATGGGTGTGTG | *CIT1* KO confirmation  (first copy) |
| CIT1-CHR | AGTAGTTTGGATGGGAACG |  |
| CIT1-orf-F | TGAAGAACCATTACCAGAAG | *CIT1* KO confirmation  (second copy) |
| CIT1-orf-R | CTAAACCATTCAAACCAGC |  |
| ACO1-L Fwd | AGTTGATTGGCATTGTGTTG | Fusion PCR for *ACO1* KO |
| ACO1-L rev | CACGGCGCGCCTAGCAGCGGAAGTCCTGCATAATTGAGATTC |  |
| ACO1-R Fwd | GTCAGCGGCCGCATCCCTGCTGCGTTCTATTGATTGTAAGAG |  |
| ACO1-R rev | TCAACGATAAACTGCCATATC |  |
| ACO1-CHF | TTAATTCCATTAACCAATCAGC | *ACO1* KO confirmation  (first copy) |
| ACO1-CHR | AAGAACTGAGATAGCCTGTC |  |
| ACO1-orf-F | TGGCTGGTATTACTACTGTC | *ACO1* KO confirmation  (second copy) |
| ACO1-orf-R | ATCTTTGATGATAGAGGCAG |  |
| ACO2-L Fwd | TTGTGAGTAAGTAGTGGAAGG | Fusion PCR for *ACO2*KO |
| ACO2-L rev | CACGGCGCGCCTAGCAGCGGCTGATCTAGCTCTTAACATTG |  |
| ACO2-R Fwd | GTCAGCGGCCGCATCCCTGCTTCTGCCATCAACTACATCG |  |
| ACO2-R rev | TCATTTCTCAAGGAGCCTAC |  |
| ACO2-CHF | TCTGAAAAGTACAACGATCC | *ACO2* KO confirmation  (first copy) |
| ACO2-CHR | AGAAGCATTGTCTACAGAAG |  |
| ACO2-orf-F | TGACATGTGGTATGAGTTCC | *ACO2* KO confirmation  (second copy) |
| ACO2-orf-R | TAACCAGTACCTCCTCTGAC |  |
| IDH1-L Fwd | AATGCCTCTATGAACACAAC | Fusion PCR for *IDH1*KO |
| IDH1-L rev | CACGGCGCGCCTAGCAGCGGTGAATAAGCTCTTGGTATCC |  |
| IDH1-R Fwd | GTCAGCGGCCGCATCCCTGCATCAACCTGAGAGATGTCAGG |  |
| IDH1-R rev | TCGATATGCGTGAGTCAGTAC |  |
| IDH1-CHF | TTCTCTCGGTACCGACAAAG | *IDH1* KO confirmation  (first copy) |
| IDH1-CHR | TTTGAAAATGTGGGTTGGTG |  |
| IDH1-orf-F | TTAATGCAAGTCGATCGTTG | *IDH1* KO confirmation  (second copy) |
| IDH1-orf-R | AAAGATTCGACAGCTTCGTC |  |
| IDH2-L Fwd | ATAACTTCCATGAGTTCCAC | Fusion PCR for *IDH2*KO |
| IDH2-L rev | CACGGCGCGCCTAGCAGCGGTAAGATTTCGGAGAGAGGAG |  |
| IDH2-R Fwd | GTCAGCGGCCGCATCCCTGCTACCACTACACGTTTCACTG |  |
| IDH2-R rev | ATGATCCACAGAGTCATTGC |  |
| IDH2-CHF | AACTCCAAGCTCACAAATAC | *IDH2* KO confirmation  (first copy) |
| IDH2-CHR | TCACCAAGTGTTTTACAACC |  |
| IDH2-orf-F | TCAGCTCCAGTTATCAGAAC | *IDH2* KO confirmation  (second copy) |
| IDH2-orf-R | TTAATGGATTGGACAACACC |  |
| KGD1-L Fwd | TACACCAATTCACAACTCGTC | Fusion PCR for *KGD1* KO |
| KGD1-L rev | CACGGCGCGCCTAGCAGCGGTAGTTTAAAGTCTCTCGGTGG |  |
| KGD1-R Fwd | GTCAGCGGCCGCATCCCTGCAGAAACCTTTCGTCAATAATCG |  |
| KGD1-R rev | AAAAGTACTGGTAACACACTACAGG |  |
| KGD1-CHF | AGACGTATTCTATTCCTGGTC | *KGD1* KO confirmation  (first copy) |
| KGD1-CHR | AAGAAGAAGGTAACCTCAAC |  |
| KGD1-orf-F | ATCCAATTCCAACTATGTCG | *KGD1*KO confirmation  (second copy) |
| KGD1-orf-R | TGGTTGAGGAACTTCAATTC |  |
| KGD2-L Fwd | TCTATGATGATTCCCAACTC | Fusion PCR for *KGD2* KO |
| KGD2-L rev | CACGGCGCGCCTAGCAGCGGGTGATGAATTACCAGAGTGG |  |
| KGD2-R Fwd | GTCAGCGGCCGCATCCCTGCCAAACAAGTACAAAGGAGAG |  |
| KGD2-R rev | AGCTTTGTTACCCTCAGAATG |  |
| KGD2-CHF | TACCAAGCAGTAATACACCTAC | *KGD2* KO confirmation  (first copy) |
| KGD2-CHR | AACTTCCATTTGTCGTGTTC |  |
| KGD2-orf-F | AACACCATTCTTGACTTTCC | *KGD2*KO confirmation  (second copy) |
| KGD2-orf-R | TAAGACGTTCAGCAATTCTC |  |
| LSC1-L Fwd | TTATAGAGGCACAAGATGTTG | Fusion PCR for*LSC1* KO |
| LSC1-L rev | CACGGCGCGCCTAGCAGCGGTGTGAGGAATGAAATGAGG |  |
| LSC1-R Fwd | GTCAGCGGCCGCATCCCTGCCTCCAGCTAGATTGGGTAAC |  |
| LSC1-R rev | TATAGTCGTGCAACATACTCC |  |
| LSC1-CHF | GTCCCTATCAATATTCAATGG | *LSC1* KO confirmation  (first copy) |
| LSC1-CHR | TATGGCTGTTTTATCCACAC |  |
| LSC1-orf-F | ACTGGTATCTTTGTTCCACC | *LSC1* KO confirmation  (second copy) |
| LSC1-orf-R | AACAGGTTTAGGACCTTCTG |  |
| LSC2-L Fwd | TTCTGTAGTGAATTCTTGACC | Fusion PCR for*LSC2* KO |
| LSC2-L rev | CACGGCGCGCCTAGCAGCGGCCTGGTGGTGTTAATGTATG |  |
| LSC2-R Fwd | GTCAGCGGCCGCATCCCTGCAACGAGAAATAGCTATCCAAG |  |
| LSC2-R rev | TACCTTCTGGTGAAAAAGTG |  |
| LSC2-CHF | AACCTGTGAGCCTAACTTTC | *LSC2* KO confirmation  (first copy) |
| LSC2-CHR | ATACCTATGGCAGTAGATCC |  |
| LSC2-orf-F | AACAGATACACACATACCTCG | *LSC2* KO confirmation  (second copy) |
| LSC2-orf-R | AACACCACCTTGTAAACCTG |  |
| SDH2-L Fwd | TGTCCCAAACAGAAGTAAAC | Fusion PCR for *SDH2* KO |
| SDH2-L rev | CACGGCGCGCCTAGCAGCGGAGGAAAAGTTAGACTTGGTG |  |
| SDH2-R Fwd | GTCAGCGGCCGCATCCCTGCAAGCCACAACCAACAAAAAC |  |
| SDH2-R rev | ATTGGACGTGTCTTTACCAG |  |
| SDH2-CHF | TCTGTAGATGTTTGACAACCAG | *SDH2* KO confirmation  (first copy) |
| SDH2-CHR | TGATATAAGACGAGCAGTACTTG |  |
| SDH2-orf-F | AAGATCCTTAGCTACTGCAG | *SDH2* KO confirmation  (second copy) |
| SDH2-orf-R | TAGAACAACAAGCACACAAG |  |
| SDH3-L Fwd | TCTACACAAAGCTTCGAGAG | Fusion PCR for *SDH3* KO |
| SDH3-L rev | CACGGCGCGCCTAGCAGCGGAATTGTTTGAGTGAGTGAGG |  |
| SDH3-R Fwd | GTCAGCGGCCGCATCCCTGCAACGTACCTTTACATGACAG |  |
| SDH3-R rev | CTCCAGTAATGTGAATAACG |  |
| SDH3-CHF | ACAAAGATGATCGAAGAACC | *SDH3* KO confirmation  (first copy) |
| SDH3-CHR | AGTTCAAATCCATGGCAATG |  |
| SDH3-orf-F | ATACACATCAACCGTACCAG | *SDH3* KO confirmation  (second copy) |
| SDH3-orf-R | ACTTCCAATGACAGCAGTAG |  |
| SDH4-L Fwd | ATTTGAACCAAGAGCAAGAG | Fusion PCR for *SDH4* KO |
| SDH4-L rev | CACGGCGCGCCTAGCAGCGGGGAGGCAGAGAATTTCTTTG |  |
| SDH4-R Fwd | GTCAGCGGCCGCATCCCTGCAGAAAGAGCTATCGAGTTGTC |  |
| SDH4-R rev | TGGTGGTGAAGGATCTATTG |  |
| SDH4-CHF | ATATCCCTTGGTATGATCGAG | *SDH4* KO confirmation  (first copy) |
| SDH4-CHR | TTAATCATCAATGGTGGTGG |  |
| SDH4-orf-F | AACTATTCCTCAACCACCAG | *SDH4* KO confirmation  (second copy) |
| SDH4-orf-R | ATATTGGAAACACCACCTTC |  |
| FUM11-L Fwd | TAATTGGATTGGTGCAAGAG | Fusion PCR for *FUM11* KO |
| FUM11-L rev | CACGGCGCGCCTAGCAGCGGTCAGATTCAATACGTGACATG |  |
| FUM11-R Fwd | GTCAGCGGCCGCATCCCTGCAACTTCAGAAGAGTTTGACG |  |
| FUM11-R rev | TACCAAGCCTTATCTAGAGG |  |
| FUM11-CHF | TAGCACATGAGATTGGGTTC | *FUM11* KO confirmation  (first copy) |
| FUM11-CHR | AAGATAAAGGAAAGTCTGTCC |  |
| FUM11-orf-F | TCATCTGCTATTCAAGAAGC | *FUM11*KO confirmation  (second copy) |
| FUM11-orf-R | TTGGTGTTTAAACCTGTTCC |  |
| FUM12-L Fwd | TGCAACTGAAAAGAGATTGG | Fusion PCR for *FUM12* KO |
| FUM12-L rev | CACGGCGCGCCTAGCAGCGGTCGTTAGGTATACTGGGTTGG |  |
| FUM12-R Fwd | GTCAGCGGCCGCATCCCTGCAAGAGCCATAAGTAGGTCACG |  |
| FUM12-R rev | TCGCTTTTCCTTCAATCTAG |  |
| FUM12-CHF | AAGACACATAAAGCATCACG | *FUM12* KO confirmation  (first copy) |
| FUM12-CHR | TTTCCCTAAGTTCTCTTTCC |  |
| FUM12-orf-F | TTAAAGCTGTTGCTCCTTTG | *FUM12*KO confirmation  (second copy) |
| FUM12-orf-R | AAAGAGTCACGTAACTTGGTC |  |
| MDH1-1-L Fwd | AATCACACATTCGCATGTAG | Fusion PCR for*MDH1-1* KO |
| MDH1-1-L rev | CACGGCGCGCCTAGCAGCGGAGAAGAAAAGGAAAGGAAAGG |  |
| MDH1-1-R Fwd | GTCAGCGGCCGCATCCCTGCAAAAAGGAGGGTTTGGATAC |  |
| MDH1-1-R rev | TGTCGTTGTTCCTGTTAGTG |  |
| MDH1-1-CHF | GAATTAAAAAGCAGGAGGTTG | *MDH1-1* KO confirmation  (first copy) |
| MDH1-1-CHR | ACTAAAAGCTTGACCCATTG |  |
| MDH1-1-orf-F | TCTTTACGATATCAGAGGTG | *MDH1-1*KO confirmation  (second copy) |
| MDH1-1-orf-R | TTAGCTTGGACAACTTCATC |  |
| MLS1-L Fwd | ACTATGACGACATTCTGACTC | Fusion PCR for*MLS1* KO |
| MLS1-L rev | CACGGCGCGCCTAGCAGCGGTGTTGGCTTCCTTCTTTGTTG |  |
| MLS1-R Fwd | GTCAGCGGCCGCATCCCTGCTTCTCGTAAGTATTTGGCTGC |  |
| MLS1-R rev | AACTCAGCGTAGTCATTGAAC |  |
| MLS1-CHF | TGATGCACGAACATGACTTC | *MLS1* KO confirmation  (first copy) |
| MLS1-CHR | ACCTGTTTGTCGTTATCAGTC |  |
| MLS1-orf-F | TGTGAAATCACTGGTCCAAC | *MLS1*KO confirmation  (second copy) |
| MLS1-orf-R | AATCCCATCTACCACAGTTC |  |
| MDH1-3-L Fwd | TTGTACTAGTGATTCCAAGC | Fusion PCR for *MDH1-3*KO |
| MDH1-3-L rev | CACGGCGCGCCTAGCAGCGGACCTGCAACAGTAACTTTGAC |  |
| MDH1-3-R Fwd | GTCAGCGGCCGCATCCCTGCAGGTACAGAATTCGTCAATG |  |
| MDH1-3-R rev | CAGCATTTCAAGAATCAGTC |  |
| MDH1-3-CHF | AGAGTGGTATAGGAATGAACTCC | *MDH1-3* KO confirmation  (first copy) |
| MDH1-3-CHR | TATGGCACAAACCAACTTTC |  |
| MDH1-3-orf-F | AACATTGCTAGAGTTGCTCC | *MDH1-3*KO confirmation  (second copy) |
| MDH1-3-orf-R | ATTCTTTACCACCTGAGACTC |  |
| PCK1-L Fwd | TATGGGCCCCGTCAACTCAAGTCCTAACC | Fusion PCR for*PCK1*KO |
| PCK1-L rev | CACGGCGCGCCTAGCAGCGGCTCGAGCAAGGGAATTTGATGGTGG |  |
| PCK1-R Fwd | GTCAGCGGCCGCATCCCTGCCCGCGGACACCCAGAATAACTGAACTC |  |
| PCK1-R rev | TACGAGCTCTTGACAATGCTTGTATGCTGC |  |
| PCK1-CHF | CATCTGCTCATTACCCGAATG | *PCK1* KO confirmation  (first copy) |
| PCK1-CHR | TCTGCAAGCTCTAGTCACAC |  |
| PCK1-orf-F | GTAAGACCACTTTGTCTGCTG | *PCK1*KO confirmation  (second copy) |
| PCK1-orf-R | ACACCGGAAGCATCACATG |  |
| PYC2-L Fwd | TATGGGCCCTCTTGATGAGGGTGAACGTTG | Fusion PCR for*PYC2*KO |
| PYC2-L rev | CACGGCGCGCCTAGCAGCGGCTCGAGGAGGTAGAAGAAAGGTATGT |  |
| PYC2-R Fwd | GTCAGCGGCCGCATCCCTGCCCGCGGCGAATGGGATTAGTTTAACG |  |
| PYC2-R rev | TACGAGCTCTCTTCTGCAGTTGTGGTTGTTG |  |
| PYC2-CHF | CCCTCTGTCTCTCTGTCTCTC | *PYC2* KO confirmation  (first copy) |
| PYC2-CHR | GTAGTGGTGGTGGTGGTAG |  |
| PYC2-orf-F | GATTCTGTGGGTGATAAAG | *PYC2*KO confirmation  (second copy) |
| PYC2-orf-R | TGGCATCACGAACCAGTTTAG |  |
| CaLEU2-FWD | ATATCTGCAGTGTTGTCAATTCCATCTACC | For construction of the reconstituted plasmids |
| CaLEU2-REV | AATCTTAAGCTTATCGTGTTGTTGATGTTTCC |  |
| CIT1-COMP-F | ATATCTGCAGTTGTACCTTTGTATCCGAATG |  |
| CIT1-COMP-R | ATATCTGCAGTTAGGAACACTGTTGCAACC |  |
| ACO1-COMP-F | ATATCTGCAGAACAGAGCGTGTGAATTGAG |  |
| ACO1-COMP-R | ATATCTGCAGAACACTAGCACTCCACAAATG |  |
| ACO2-COMP-F | AATCTTAAGCTTACAACAGAACCAAGGTGTAAG |  |
| ACO2-COMP-R | AATCTTATCGATTATGACCAGAAAAGATCACG |  |
| IDH1-COMP-F | AATCTTAAGCTTTCCCTGTTCACTTTTCTTTTC |  |
| IDH1-COMP-R | AATCTTATCGATATACGACGTCACACATGATTC |  |
| IDH2-COMP-F | AATCTTAAGCTTTGACGATTCACTGATAACACG |  |
| IDH2-COMP-R | AATCTTATCGATAGCAACGCAGAGAACAATTC |  |
| KGD1-COMP-F | ATATCTGCAGAATACAAGGATCGGAGTTCAC |  |
| KGD1-COMP-R | ATATCTGCAGAGGTAGAATGGTTTGCATCAG |  |
| KGD2-COMP-F | ATATCTGCAGATCAATGTAGGTTAGAGCTCAC |  |
| KGD2-COMP-R | ATATCTGCAGTGCTAATCATGTGTTCCAAC |  |
| SDH2-COMP-F | AATCTTATCGATAAGAAAGCTATGTGGTGGTG |  |
| SDH2-COMP-R | AATCTTATCGATTTGCAGTATCTCTCAATCCAC |  |
| SDH3-COMP-F | ATATCTGCAGAACAAAGATGATCGAAGAACC |  |
| SDH3-COMP-R | ATATCTGCAGAATGACATGAGTGGCTCCAG |  |
| SDH4-COMP-F | ATATCTGCAGTGATATCCCTTGGTATGATCG |  |
| SDH4-COMP-R | ATATCTGCAGTGGTGGTGAAGGATCTATTG |  |
| FUM11-COMP-F | ATATCTGCAGAAACACTTGACTACACACACCAC |  |
| FUM11-COMP-R | ATATCTGCAGTTTGCCTCTGGAGAAACAAG |  |
| FUM12-COMP-F | ATATCTGCAGTTGCTCCTGAAGTATGATTAC |  |
| FUM12-COMP-R | ATATCTGCAGAGATCTGGTTGAAGTAGATGAC |  |
| MDH1-1-COMP-F | ATATCTGCAGTGAGATTGACATGCAGAATTC |  |
| MDH1-1-COMP-R | ATATCTGCAGTTTACCAGCACCATCTAGACC |  |
| MLS1-COMP-F | ATATCTGCAGACAGTTGATGTCTTTTGTTGC |  |
| MLS1-COMP-R | ATATCTGCAGTATTCGAGATCGCAAATGAC |  |
| MDH1-3-COMP-F | AATCTTATCGATTTCCAAGTGACGTTTGTTTG |  |
| MDH1-3-COMP-R | AATCTTATCGATTTTCCTCTTTTCCAGTCAGG |  |
| CIT1-F | TTGTTGACTGGTGAAGTTCC | For Q-RT-PCR |
| CIT1 -R | TCAGATTCCAAAGCAGTAACG |  |
| ACO1 -F | TGACTTGAACACCTTGGAAC |  |
| ACO1 -R | TCTTCATATGAGGAGTTGGTAC |  |
| IDH1-F | TCACTCCTAACTTGTACGGTTC |  |
| IDH1 -R | ATCTAAACCAACGTGACGAC |  |
| IDH2 -F | TGTCACTCCATTGTTGATTG |  |
| IDH2 -R | TCTCAAAGTCAAGTTCATGG |  |
| KGD1 -F | AACTTACTGCTCCTCCTATGG |  |
| KGD1 -R | TTTGGGAACTTACTTGACAAG |  |
| KGD2-F | ATCGATGTTGAAGTCAATGC |  |
| KGD2 -R | TTCTTCTTTCTTAGCTGGAGC |  |
| SDH2 -F | TCGTGAAGGTATTTGTGGTTC |  |
| SDH2 -R | TCTCTGACAACGAACATGTG |  |
| SDH3 -F | TCAACCGTACCAGCAACTTC |  |
| SDH3 -R | AGTAGCAGCAAATCCACAAG |  |
| MDH1-1-F | TTTCCTCTTCTGCTTCCAAC |  |
| MDH1-1-R | ACCTTTGACAGTGGAGTTAG |  |
| HWP1-F | AAAATCAGATGTTCCAGCTAC |  |
| HWP1-R | TGGTTTAGTTTCAGTACCAGC |  |
| ECE1-F | ACCATGCTCCAGAATTCAAC |  |
| ECE1-R | TGGATTACTTGTGGAATGTTG |  |
| RAS1-F | ATCAAGATGGATTAGCATTGG |  |
| RAS1-R | TGTTGTTGCTGTTGTTGTTG |  |
| CYR1-F | AGAAAGAAGACGATGAAACAG |  |
| CYR1-R | AGGAGAACTAGAGGATGTAGAC |  |
| TPK1-F | AGAACTTGCCAACAAACAAC |  |
| TPK1-R | TTTCTTGGTCAAGGAAAGAC |  |
| TPK2-F | TTGTTGCCTGAACGTTCTAC |  |
| TPK2-R | CTACCATTGTGAACTGATCTC |  |
| EFG1-F | ACAACCTCAGCATTACAATG |  |
| EFG1-R | ATAGGTACTGCTTGTTGACC |  |
| FLO8-F | AGCAAATGACTAAGATGGCTG |  |
| FLO8-R | AGTCGGAATTACCAGTGTTTC |  |
| BRG1-F | TGTCAAAGTGCAACCTCAAC |  |
| BRG1-R | ATTGGTGAAGATTGTTGTTGG |  |
| NDT80-F | AATCGTCGACCAGAAAAGTC |  |
| NDT80-R | ATGCAACAATGGTGTCACTG |  |
| SFL2-F | TCGACAACTTCACATGTATG |  |
| SFL2-R | TTGAATCTTCCACTCAGATG |  |
| ICL1-F | AAACACGATGCTGACAAGAC |  |
| ICL1-R | ATCTGGAGATGGTTCGTTAG |  |
| MLS1-F | TCCAACGTTGATTGTGAGAC |  |
| MLS1-R | AAAGTATGGACCAAAGCCTC |  |
| PYC2-F | TGTTCATGTTCAGGGTCTAC |  |
| PYC2-R | AGTAGTCCAACAATCACCAG |  |
| ACT1-F | TAAGATTATTGCTCCACCAG |  |
| ACT1-R | ACCAGATTCGTCGTATTCTTG |  |
| PDE2-L Fwd | TCTTTTCCACTCAGTTCAACC | Fusion PCR for *PDE2*  first copy KO |
| PDE2-L rev | CACGGCGCGCCTAGCAGCGGAATCTCGAGGTCAACCAATG |  |
| PDE2-R Fwd | GTCAGCGGCCGCATCCCTGCAACTAGACCAAGAAGTTTGC |  |
| PDE2-R rev | ATACAAAGCTCCCAGACAAC |  |
| PDE2-CHF | ACTTGACACAAAACTTGGAG | *PDE2* KO confirmation  (first copy) |
| PDE2-CHR | TATACACACCACCTTGTTGC |  |
| PDE2-KO-F | AGTATCTACTGATTTGTTTGGTTTACCATTCCTTTAAATATACACCAACAAAATAGATATTTACCGGGCCCCCCCTCGAGGAAGTT | PCR for *PDE2*second copy KO |
| PDE2-KO-R | AAACTTCTTGGTCTAGTTTAACTTAACTGCAAGCATACACACAGTGAAACACTACATTGGCCGCTCTAGAACTAGTGGATC |  |
| PDE2-orf-F | AAATTGGACAGGAACAACTC | *PDE2* KO confirmation  (second copy) |
| PDE2-orf-R | TATGTGTGTCATGAACTTCG |  |
| SFL2-F | ATATGATATCATGAGTAAGAAAAATCCTGGTG | For construction of MYC-tagged Sfl2 strain |
| SFL2-R | CGTTAATTAACCCGGGGATCCGTTTATTCATATTATCAGTATCATC |  |
| MYC-F | CGGATCCCCGGGTTAATTAACG |  |
| MYC-R | AAGGTACCCGCGAATTCACTAGTGATTGATTAA |  |
| CIT1-F-1 | AAGCTTTGTACCTTTGTATCC | For ChIP-PCR |
| CIT1-R-1 | TGGGTTTAGTTTTGTATGCAC |  |
| CIT1-F-2 | AGTGGTGTAGTTTTCATTACC |  |
| CIT1-R-2 | ACCAGCATCACGTTTATTC |  |
| CIT1-F-3 | TTTTTGCTCTCCATCTAGTG |  |
| CIT1-R-3 | TTAGTTTTGTGTCTGTCGTG |  |
| CIT1-F-4 | TTCACCACAACTTCAAGAGC |  |
| CIT1-R-4 | AGATGGGAAAAGGAAAAGAAG |  |
| SDH2-F-1 | AATCACCAATTGTCCCAAAC |  |
| SDH2-R-1 | ACACCACCACATAGCTTTC |  |
| SDH2-F-2 | AGTGTTACTACTATAGTTGCTG |  |
| SDH2-R-2 | TCTGCTCTGTTAAGTGTATG |  |
| IDH1-F-1 | TGAATGGAACTAAGTTGTGG |  |
| IDH1-R-1 | TTTTCTTGAAGGCAGAGGAG |  |
| IDH1-F-2 | TCTGCCTTCAAGAAAATGC |  |
| IDH1-R-2 | ATGCTGTGAATAAGCTCTTGG |  |
| IDH2-F | TCTTCTTTGGTTGTTGCATG |  |
| IDH2-R | TGTTGTAGATGAGAGAGAGAGAGAG |  |
| MDH1-1-F | ACGAAATCTGGAGTATGAAC |  |
| MDH1-1-R | TGTTGAGCAAACTGAATCAC |  |
| SFL1-F | AAGAAAAAAGGTCAGGGAGAG |  |
| SFL1-R | TGCAAGACCACATGAAGTTG |  |
| EFG1-F | ACTTGGTTCAAAGAAAGGAACG |  |
| EFG1-R | TTAGGTTGTAGTGAAAGCTTGC |  |
| NRG1-F | TATACCTAGTAGCTGCATCC |  |
| NRG1-R | TTCAACAATGGTGGAGAATG |  |
